# Overexpression of *Lilium formosanum* *MADS-box* (*LFMADS*) Causing Floral Defects While Promoting Flowering in *Arabidopsis thaliana*, Whereas Only Affecting Floral Transition Time in *Nicotiana tabacum*

**DOI:** 10.3390/ijms19082217

**Published:** 2018-07-29

**Authors:** Wan-Yu Liao, Lee-Fong Lin, Ming-Der Lin, Sheng-Che Hsieh, Althea Yi-Shan Li, Yueh-Shiah Tsay, Ming-Lun Chou

**Affiliations:** 1 Institute of Medical Sciences, Tzu-Chi University, Hualien 97004, Taiwan; 98751101@gms.tcu.edu.tw; 2 Department of Life Sciences, Tzu-Chi University, Hualien 97004, Taiwan; leelin@gms.tcu.edu.tw (L.-F.L.); schsieh0513@gmail.com (S.-C.H.); 103711117@gms.tcu.edu.tw (A.Y.-S.L.); 3 Department of Molecular Biology and Human Genetics, Tzu-Chi University, Hualien 97004, Taiwan; mingder@gms.tcu.edu.tw; 4 Division of Crop Improvement, Hualien District Agricultural Research and Extension Station, Council of Agriculture, Executive Yuan, Hualien 97365, Taiwan; ystsay@mail.hdais.gov.tw

**Keywords:** transcriptome analysis, MADS-box genes, *Lilium formosanum*, floral transition, floral organ identity

## Abstract

The Formosa lily (*Lilium formosanum*) is one of the most common horticultural species in Taiwan. To explore gene regulation involved in this species, we used transcriptome analysis to generate PH-FB (mixed floral buds) and PH-LF (mature leaves) datasets. Combination of the PH-FB and PH-LF constructed a de novo assembly of the ALL dataset, including 18,041 contigs and 23,807 unigenes by Nr, GO, COG, and KEGG databases. The differential gene expression (DGE) analysis revealed 9937 genes were upregulated while 10,383 genes were downregulated in the developing floral buds compared to mature leaves. Seven putative genes (*LFMADS1 to 7*) encoding floral organ identity proteins were selected for further analysis. *LFMADS1-6* genes were specifically expressed in the floral organ, while *LFMADS7* in the floral buds and mature leaves. Phylogenetic analysis revealed that LFMADS1-3 is classified into B-class, LFMADS4 into C-class, LFMADS5 into D-class, and LFMADS6-7 into E-class, respectively. LFMADS-GFP fusion proteins appeared to localize in the nucleus, supporting their roles as transcription factors (TFs). Overexpression of the *LFMADS2*, *LFMADS4*, and *LFMADS6* genes in *Arabidopsis* resulted in early flowering and floral defect, however, only early flowering in transgenic tobacco was observed. Highly expressed floral integrator genes, including *AtFT*, *AtLFY*, and *AtFUL* in transgenic *Arabidopsis* and *NtFUL* and *NtSOC1* in transgenic tobacco, resulted in early flowering phenotype through qRT-PCR analysis. Yeast two-hybrid analysis suggested that LFMADSs may form higher order complexes with the B-, C-, D, and/or E-class proteins to determine the floral organ identity. Furthermore, E-class LFMADS proteins may function as a glue to mediate and strengthen the protein-protein interactions. Therefore, our de novo datasets would provide information for investigating other differentially expressed candidate transcripts. In addition, functional conservation of LFMADSs appears to be vital in floral transition and floral organ identity.

## 1. Introduction

Flowering functions as a switch from vegetative to reproductive growth in angiosperms, through which shoot apical meristems turn into floral meristems and then develop as floral organs [1,2]. Five major pathways in flowering process were well-known through forming complex networks between exogenous and endogenous signals, including the photoperiod pathway, the autonomous pathway, the gibberellin pathway, the vernalization pathway, and the thermosensory pathway in *Arabidopsis thaliana* [3,4,5,6]. A set of floral pathway integrator genes such as *Flowering locus T* (*FT*), *SUPPRESSOR OF OVEREXPRESSION OF CONSTANS1* (*SOC1*), and *LEAFY* (*LFY*) are located in the downstream of the floral pathways and are reported to be associated with the final steps of floral organ development [7,8,9,10,11]. The MADS (*MCM1*/*AGAMOUS*/*DEFICIENS*/*SRF*)-box transcription factor (TF) family genes were shown earlier to play crucial roles in controlling plant and animal development [12]. These TFs have been classified into two types (type I and type II) based on sequence relationships and structural features [13]. In the four-whorled flower of *Arabidopsis*, type II MADS box genes (MIKC-type) work together to specify the identity of floral organs [14,15]. Type II MADS domain containing proteins consist of the N-terminal MADS (M) domain involved in DNA binding, the Intervening (I) domain that specifies dimerization, a Keratin (K) domain mediated in protein–protein interactions, which possibly functions through the formation of coiled coils, and a C-terminus (C) containing an activation domain involved in higher order complex formation [16,17]. In *Arabidopsis*, 107 MADS-box genes have been reported previously, including 39 of the MIKC-type [18], whereas 75 genes have been annotated in rice, of which 38 are MIKC-type [19]. Notably, the potential functional role of MADS-box TFs in the regulation of flower formation is still not clear.

The ABCDE model revealed that MADS-box genes fall into different categories based on their spatial–temporal function in floral development [20,21,22]. The expression of A-class genes [*APETALA1* (*AP1*) and *APETALA2* (*AP2*)] were reported to drive the development of sepals alone (whorl 1) [23], and together with the expression of B-class genes [*APETALA3* (*AP3*) and *PISTILLATA* (*PI*)] forming petals (whorl 2). The expression of C-class genes [*AGAMOUS* (*AG*)] alone determines the development of carpels (whorl 4), and together with the expression of B-class genes generating stamens (whorl 3). D-class genes [*SEEDSTICK* (*STK*), *SHATTERPROOF1* (*SHP1*), and *2* (*SHP2*)] are involved in ovule formation [14,15,22], whereas E-class genes [*SEPALLATA* (*SEP1*, *SEP2*, *SEP3*, and *SEP4*)] are necessary for the correct development of all floral organs [24]. It is of interest to note that all except *AP2* gene of the A-, B-, C-, and E-class genes characterized in plants are the MADS-box genes [17]. In addition, MADS-box proteins form multimeric protein complexes consisting of four proteins that determine the identity of floral organ primordia according to the Floral Quartet Model [25]. The ABCDE model, initially developed in *A. thaliana*, works well for most eudicots. However, slight modifications are required to fit into the floral organ formation in certain monocots, such as lilies and orchids. In these two species, sepals and petals show similar morphology and are together termed tepals. The expansion of B-class genes expression to whorl 1 was thought to determine the presence of sepaloid petals (tepals) in these plant groups [26,27].

Recently, high-throughput transcriptome sequencing and digital gene expression tag profiling have become robust tools for identifying the novel genes involved in specific biological pathways to characterize non-model organisms without a reference genome resource [28,29,30]. Lilies are monocots, which include numerous species with various patterns of shape and color in their bulbous flowers. They belong to the most crucial horticultural and ornamental plants in the cut-flower market around the world. Several MADS-box genes have been recently identified in lily, however, most research is limited in the investigation of Easter lily (*L. longifilorum*), which is endemic in Taiwan [31,32,33,34,35,36,37,38,39]. *L. formosanum*, also known as the Formosa lily, with showy, trumpet-shaped flowers is closely related to the Easter lily, which displays a marked geographic cline and shows high resistance to drought, pathogens and climatic fluctuations [40]. Previous studies involved in regulating lily flowering time were mainly focused on the vernalization pathway [41,42,43,44] and photoperiod pathway [45,46]. Several genes have been identified in Liliaceae family and their potential biological functions related to the flowering time control, such as *flowering locus T* (*FT*) homologs *LlFT* in *L. longiflorum* [47], *LfFT1* in *L.* x *formolongi* [48], and eight *CONSTANS-LIKE* (*COL*) family members (*LfCOLs*) in *L.* x *formolongi*. [48]. Using *L.* x *formolongi* transcriptome datasets from four developmental stages, including vegetative juvenile, flowering induction (I and II), and floral differentiation analyzed the global gene expression profiles during the flowering initiation process. In total, 85 differentially expressed genes relevant to the flowering were discovered. Among these genes, members of the MADS-box, SBP-box, and CO-like transcription factor families were the most represented [49]. The MADS-box gene family, containing a highly conserved MADS domain of approximately 60 amino-acid sequences in the N-terminal region, is an important TF family that plays prominent roles throughout the life cycle of plant’s embryo to their gametophyte development [50]. As a result of a number of duplication events, more than 100 similar genes may exist in a representative genome of a flowering plant, having divergent functions of these paralogs [51,52].

In this study, we constructed and assembled transcriptome datasets from the developing floral buds of 0.5–3 cm in length (PH-FB dataset) and mature leaves (PH-LF dataset), respectively. Subsequently, an ALL dataset was generated from the combination of the PH-FB and PH-LF datasets. Extensive DGE analysis was performed to identify the differentially expressed genes and the potential metabolic pathways they may be involved. Thus, the validation of our transcriptome datasets provides information to identify unique transcripts. From the expression profiles of the DGE analysis, we selected seven *L. formosanum MADS*-box containing genes 1 to 7 (*LFMADS1* to *7*) to further study their gene expression patterns, amino acid sequence alignments, phylogenetic analysis, subcellular localization, and protein-protein interactions among proteins encoded by these genes. Our functional analysis suggested that these *LFMADSs* genes play crucial roles in regulating the floral transition and floral formation in transgenic *Arabidopsis* and transgenic *tobacco*. 

## 2. Results and Discussions

### 2.1. Illumina Sequencing, De Novo Assembly, and Functional Annotation of the L. formosanum Transcriptome

To obtain an overview of the Formosa lily transcriptome and how genes are dynamically and differentially expressed during floral transition, two Formosa lily cDNA libraries from mixed floral buds (0.5–3 cm in length) and vegetative leaves were subjected to Illumina HiSeq^TM^ 2000 sequencing. The resulting de novo transcriptomes were subsequently examined by using the bioinformatics analysis. The transcriptome datasets from floral buds and mature leaves were named as PH-FB and PH-LF, respectively. The flowchart of our transcriptome analysis is shown in Figure S1.

After removing the low-quality reads and trimming off the adapter sequences, we gained two transcriptome datasets. These datasets contain a total of 6,181,111,200 (6.18 Gb) and 6,356,535,300 (6.36 Gb) nucleotides with high-quality clean reads for PH-FB and PH-LF transcriptomes, respectively. An overview of the sequencing and assembly is given in Table S1. All high-quality reads were assembled de novo by using the Trinity program, producing 41,848 unigenes in ALL transcriptome dataset which was the combined results of the PH-FB and PH-LF transcriptome datasets. The average unigene length of 971.03 bp and N50 of 1,456 bp representing 50% of the assembled bases were incorporated into contigs of 1,456 bp or longer in this dataset (Table S2). The min length of all unigenes is 200 bp and the max length of all unigenes is 9,772 bp long. Overview of the size distribution of unigenes from ALL transcriptome dataset is shown in Figure S2. The results of species distribution showed that 36.31% of Formosa lily unigenes had top matches with those of *Elaeis* *guineensis* genes, followed by matches to *Phoenix dactylifera* (28.41%) and *Musa acumininata* subsp. *malaccensis* (10.49%) (Figure S3). These results thus indicated that our transcriptome datasets can accurately predict the unigenes potentially useful for further analysis of *Lilium* species. All the short reads were deposited in the National Center for Biotechnology Information (NCBI) and can be accessed in the Sequence Read Archive (SRA) (accession number SRX3822957 for PH-FB transcriptome dataset and SRX3822958 for PH-LF transcriptome dataset). 

A total of 31,648 unigenes (75.63% = 31,648/41,848 of ALL unigenes, Table S2) were annotated by using the BLASTX and a variety of protein databases taking into account the identity between the unigene sequences and the sequences in the database (*E*-value < 10^−5^). In addition, 30,853 (73.73%), 22,722 (54.29%), 7,060 (16.87%), 13,781 (32.93%), and 24,306 (58.08%) unigenes were aligned against the Nr, SWISS-PROT, GO (Gene Ontology), COG (Clusters of Orthologous Groups of proteins), and KEGG (Kyoto Encyclopedia of Genes and Genomes) databases, respectively. According to the GO classifications, a total of 41,848 Formosa lily unigenes with putative functions assigned to 7,060 unique sequences were categorized into three main GO categories (biological process, cellular component and molecular function) and 54 sub-categories (functional groups) (Figure S4A). When searched by using the COG database, the possible functions of 13,781 Formosa lily unigenes were predicted and classified into 25 COG categories (Figure S4B). To identify the potential biological pathways in the Formosa lily, we used KEGG program to assign total of 24,306 unigenes into 133 KEGG pathways, including “metabolism,” “genetic information processing,” “environmental information processing,” “cellular process,” and “organismal systems” in level 1 pathways. The statistics of the unigene number in each KEGG pathway is summarized in Figure S5 and Table S3. Therefore, our results give prominence to the considerable potential of using our Formosa lily transcriptome datasets to discover and further investigate the metabolic pathway genes.

### 2.2. Analysis of Differential Gene Expression in Developing Floral Buds and Vegetative Leaves in L. formosanum Transcriptome

**Figure 1:**
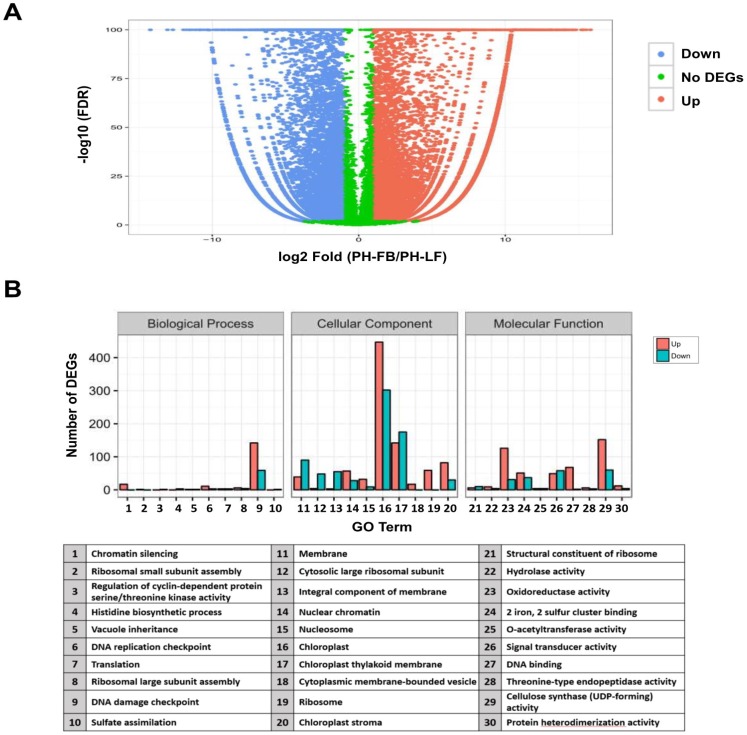

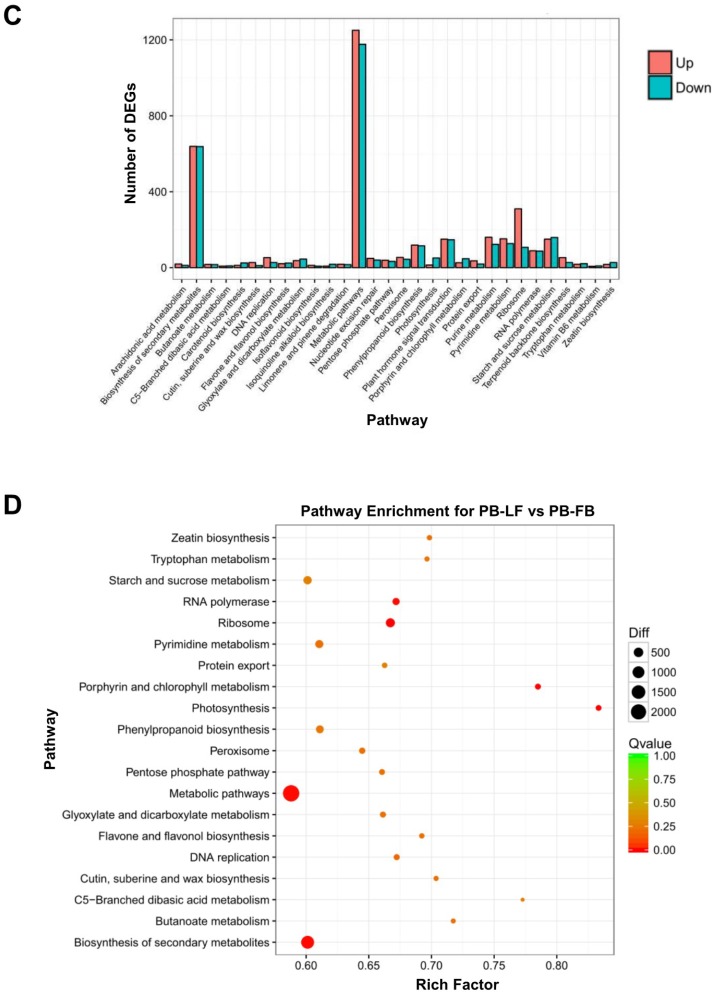
Statistics of all comparison among differentially expressed genes (DEGs) in GO and KEGG enrichment analysis. (**A**) Volcan plot for the distribution of DEGs. The differences in expression between PH-FB (developing floral buds) and PH-LF (mature leaves) were analyzed by using the FDR ≤ 0.001 and a fold change (the ratio of PH-FB/ PH-LF, log2) ≥ 2 as the criteria to screen DEGs. Each dot represents a unigene, and red and blue dots indicate DEGs that are up- and down- regulated, respectively. The green dots represent unigenes with no DEGs changes in these two samples. (**B**) Histogram of the DEGs number (up and down) in the enriched GO terms. These GO terms were classified into Biological Process, Cellular Component, and Molecular Function groups. (**C**) Histogram of the DEGs number (up and down) in the most enriched pathways. The most enriched pathways (30 terms) were selected, and statistics according to the up- and down- regulation of DEGs were compared with the controls. (**D**) The scatter plot from the results of DEGs enriched pathways. The Y-axis shows the top 20 enriched KEGG pathways. These 20 pathways were sorted with the increasing significant level from the bottom to the top on the Y-axis. The X-axis indicates the enrichment factor (the enriched gene number is proportional to the background gene number) for each enriched pathway. The larger points correspond to more DEGs numbers and the different colors of points show the different Q values.

To identify genes displaying a significant change in their expression for both reproductive floral organ and vegetative leaves, differentially expressed tags were analyzed by comparing the PH-FB (developing floral buds) library with that of the PH-LF (vegetative leaves). False Discovery Rate (FDR) ≤ 0.001 and log2 fold-change ≥ 1 were used as the threshold to assess the significance of differential gene expression. A total of 20,320 differentially expressed genes were detected between PH-FB and PH-LF libraries. The distribution of DGE pattern is represented as volcan plot (Figure 1A). The DGE analysis revealed that a total of 9937 unigenes were upregulated and 10,383 unigenes were downregulated (Figure 1A). To explore the changes in terms of the patterns of gene expression, the percentage of genes in GO categories was determined by the enrichment analysis for all differentially expressed genes (DEGs). As regards the category of biological function, the percentage of genes mapped to “translation (201 unigenes: 142 upregulated and 59 downregulated)” was significantly enriched, while in that of cellular component, the percentage of genes mapped to “integral component of membrane (749 unigenes: 447 upregulated and 302 downregulated)” was obviously increased. In addition, enrichment also occurred for genes associated with molecular function, such as “structural constituent of ribosome (212 unigenes: 152 upregulated and 60 downregulated)” and “DNA binding (157 unigenes: 126 upregulated and 31 downregulated)” (Figure 1B). In order to survey genes particularly involved in certain pathways, these differentially expressed transcripts were mapped to the KEGG pathways. In total, 13,685 genes implicated in 133 pathways were annotated by using the KEGG database, of which “metabolic pathways (2428 unigenes: 1251 upregulated and 1177 downregulated)” and “biosynthesis of secondary metabolites (1277 unigenes: 639 upregulated and 638 downregulated)” interpreted the most genes (Figure 1C, Table S4). The scatter plot also showed the metabolic pathways and biosynthesis of secondary metabolites enriched the most genes among the top 20 pathways (Figure 1D). These metabolic pathways, therefore, may interact with each other to constitute a complex floral transition regulatory network.

### 2.3. Identifying LFMADS Genes in L. formosanum Transcriptome

In order to identify MADS-box homologous genes differentially expressed in the developing floral buds and vegetative leaves of *L. formosanum*, we used MADS-box protein sequences of *Arabidopsis* and *Lilium* species retrieved from NCBI database to blast our transcriptome datasets generated in this study. As shown in Table S5, we discovered 33 unigenes interpreted as *L. formosanum* MADS-box (LFMADS) proteins. These unigenes appeared to have high homology with *MADS-box* genes isolated from other plant species annotated in Nr and SWISS-PORT protein databases.

**Figure 2:**
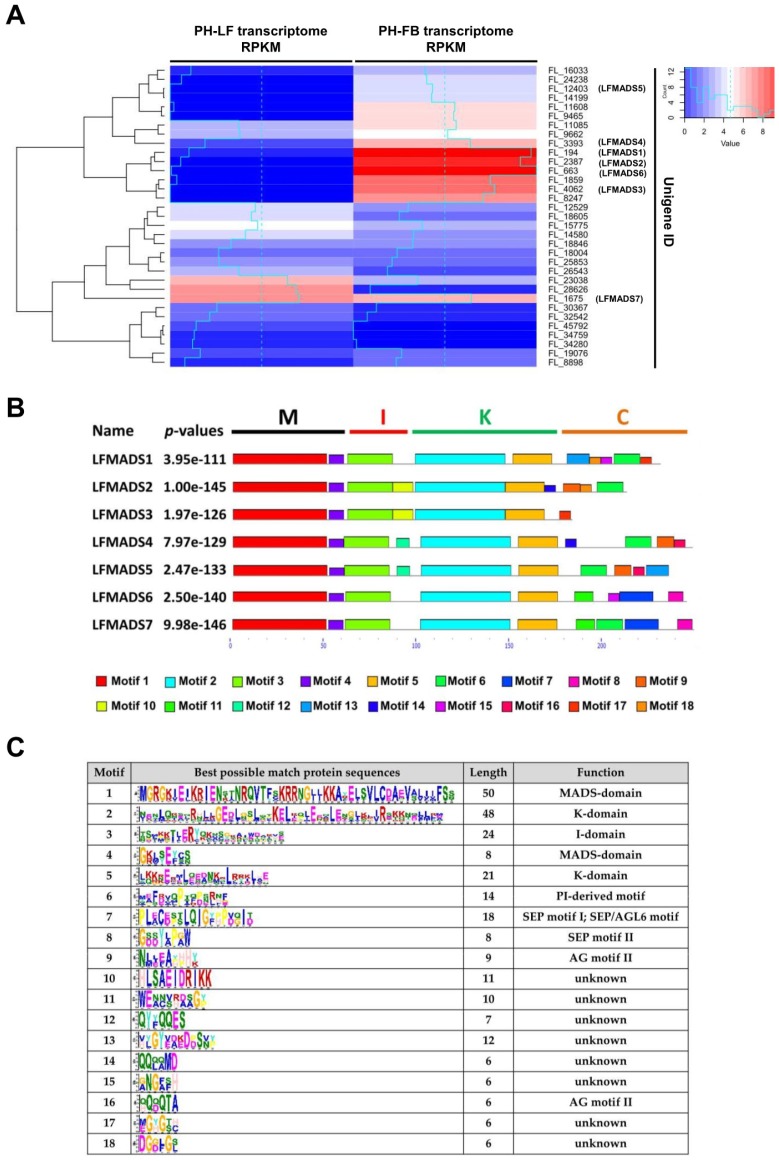
The expression profiles and motifs identified using online MEME tools for LFMADS1–7. (**A**) The expression patterns of unigenes homologous with MADS-box genes in *Arabidopsis* identified from PH-FB and PH-LF transcriptome data. The values of log_2_ [RPKM] represent the expression level for each unigene in floral buds and mature leaves, respectively. From blue to red colors in the map indicate the expression levels from low to high. (**B**) The motif location and combined *p*-value of LFMADS1–7 are shown on the left and denoted by rectangles with different colors. (**C**) Possible amino acid sequences and functions of Motifs 1–18 identified using MEME tools for LFMADSs.

In order to evaluate the *LFMADS* differential expression obtained from the developing floral buds compared to the mature leaves, we specifically selected full-length *LFMADS1-7* for further investigation not only because they are homologous to floral organ identity genes involved in the flower development and floral organ specification in *Arabidopsis*, but also to confirm that the results of our DGE analysis are reliable and thus would provide researchers with sufficient information to exam other differentially expressed genes between these two tissues. The genes characteristics and accession numbers for *LFMADSs* are listed in Table 1 and the expression profiles for these *LFMADSs* are shown in Figure 2A. The online Multiple EM for Motif Elicitation (MEME) motif search tool was subsequently used and MIKC-type MADS-box proteins’ corresponding conserved motifs in seven LFMADSs were determined (Figure 2B,C). These *LFMADS1-7* genes, identified in *L. formosanum* transcriptome and confirmed by RT-PCR with gene-specific primer sets (Table S6), indeed belong to the MIKC-type MADS gene family and we purpose that their functions may be implicated in plant floral development.

**Figure 3:**
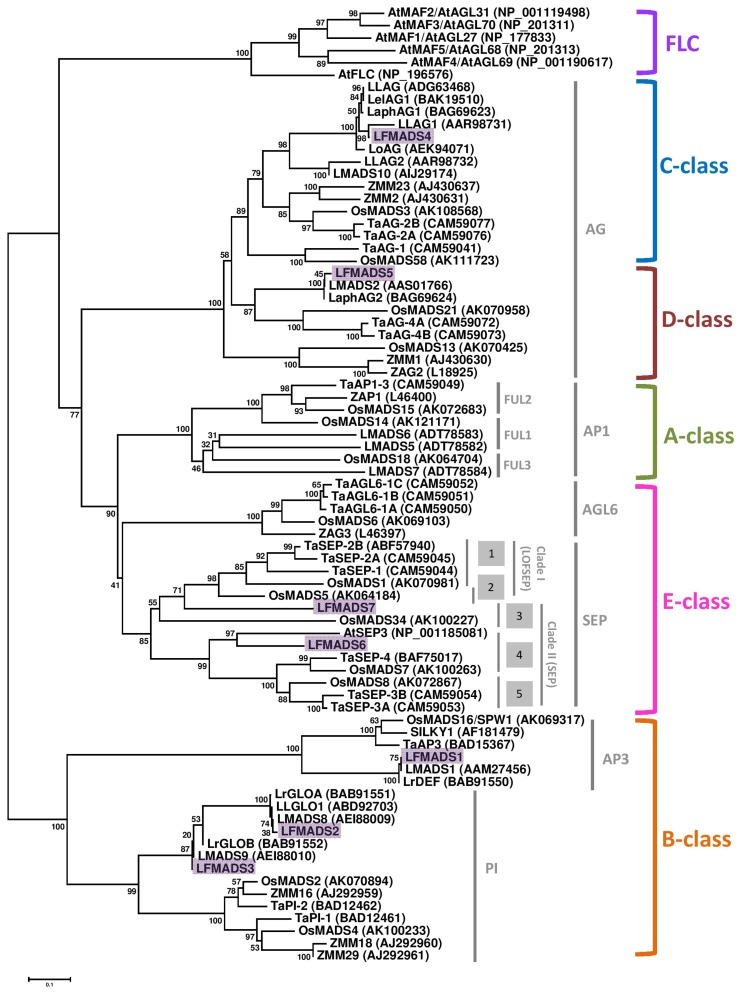
Phylogenetic tree of LFMADSs and its orthologs from various plant species. The deduced full-length amino acid sequences were used for the alignments. Proteins from *L. formosanum* were highlighted with purple boxes. Amino acid sequences of A-, B-, C-, D-, and E-class MADS-box genes were retrieved via the NCBI server (http://www.ncbi.nlm.nih.gov/). Total of 71 MADS-box proteins were adapted in this phylogenetic analysis: 7 from *L. formosanum*, 11 from *L. longiforum*, 3 from *L. regale*, 5 from *L.* hybrid cultivar, 1 from *Arabidopsis thaliana*, 16 from rice (*Oryza sativa*), 10 from maize (*Zea mays*), and 18 from wheat (*Triticum aestivum* L.). The phylogenetic tree was generated with the neighbor-joining algorithm and evaluated by bootstrap analysis (MEGA version 6.0). Numbers on major branches indicate bootstrap percentage for 1000 replicates. Subfamilies of the plant MADS-box genes and the functional classification according to the A-, B-, C-, D- and E-classes are indicated at the right margin. Six *Arabidopsis* sequences of the FLC subfamily were used as outgroups in this study.

To examine the putative functional classification of the LFMADS1 to 7 in relation to the ABCDE model and to gain some insight into the potential functions of LFMADS proteins from well-studied MADS-box proteins in other plant species, we used full-length amino acid sequences to perform a phylogenetic analysis of MADS-box proteins from *Arabidopsis*, rice, wheat, maize, and other lily cultivars (Figure 3). The phylogenetic analysis indicated that the 73 MADS-box proteins were clearly grouped into five different clades corresponding to the ABCDE model. Within each functional class, three LFMADSs (LFMADS1, LFMADS2, and LFMADS3) are classified into the B-class lineage, LFMADS4 into the C-class lineage, LFMADS5 into the D-class lineage, and two (LFMADS6 and LFMADS7) into the E-class lineage, respectively (Figure 3).

The cDNA sequences of *LFMADS1*, *LFMADS2* and *LFMADS3* encode polypeptides containing 229, 211 and 182 amino acid residues, respectively. Next, we compared the sequence similarity of LFMADSs with other lily and rice MADS-box proteins in each functional class. LFMADS1 was most closely related to other lily such as *L. longiflorum* LMADS1 (100% identity) and *L. regale* LrDEF (99.6% identity) in the AP3 clade, while only showed 68.9% identity to rice OsMADS16 (SPW1) (Figure 3; Figure S6). LFMADS2 and LFMADS3 belong to the PI family, with LFMADS2 closely related to *L. longiflorum* LLGLO (99.5% identity) and *L. regale* LrGLOA (99.5% identity), and LFMADS3 most closely related to *L. regale* LrGLOB (98.3% identity) (Figure 3, Figure S6). LFMADS2 also showed 67.5% and 63.7 identity to rice OsMADS2 and OsMADS4, respectively. Similar identity also showed in LFMADS3 to OsMADS2 (70.4%) and OsMADS4 (67.8%). The high sequence identity among LFMADS1, LFMADS2, LFMADS3 and AP3/PI orthologs suggest that *LFMADS1*, *LFMADS2*, and *LFMADS3* belong to *L. formosanum* B-class orthologs. The presence of one AP3 ortholog (LFMADS1) and two PI orthologs (LFMADS2 and LFMADS4) is similar to other monocots B-class MADS-box protein, supporting the notion that ancient paralogy in one class of floral functional genes may occur through gene duplication event. These duplications of floral homeotic genes may have played a critical role in the diversification of floral homeotic functions and thus caused the evolution of flowers.

LFMADS4 and LFMADS5 proteins belong to the AG family, which is functionally classified as a C/D class (Figure 3, Figure S7). The *LFMADS4* cDNA encodes a 255 amino acid protein which is closely related to *L. longiflorum* LLAG1 (88.6% identity) in the C-functional class, while only shares 70.3% identity with another putative C-class protein LMADS10. The LFMADS4 protein also contains two highly conserved AG motifs in its C-terminal domain identified in most C/D class proteins (Figure S7). The high sequence identity between LFMADS4 and AG orthologs suggests that *LFMADS4* belongs to *L. formosanum* C-class gene family and is closely related to *LLAG1*. On the other hand, *LFMADS5* cDNA encodes a protein with 233 amino acids shares 98.7% identity with *L. longiflorum* LMADS2, while only shows 64.7%, 63.4%, and 59.7% identity to *Triticum aestivum* TaAG-4A, TaAG-4B, and *Oryza sativa* OsMADS21, respectively. Thus, the high sequence identity between LFMADS5 and D-class orthologs suggests that *LFMADS5* is classified into *L. formosanum* D-class (Figure 3, Figure S7).

The cDNA sequences of *LFMADS6* and *LFMADS7* encode 242 and 246 amino acid proteins, respectively, and contain the typical SEP-I and SEP-II motifs (Figure S8) in the C-terminal domain that is normally identified in most SEP proteins. The LFMADS6 protein shows 68.4%, 66.9% and 64.8% identity to *O. sativa* OsMADS8, *T. aestivum* TaSEP-3A, and *O. sativa* OsMADS7, respectively. These data suggest that *LFMADS6* and *LFMADS7* belong to the *SEP* gene family and grouped as E class (Figure 3, Figure S8). The phylogenetic tree of Formosa lily and *Arabidopsis* also showed the close relationship of MADS proteins in these two species (Figure S9), which thus provides good evidences for their functional similarities.

### 2.4. Expression Patterns of Seven MADS-Box Genes of L. formosanum

**Figure 4:**
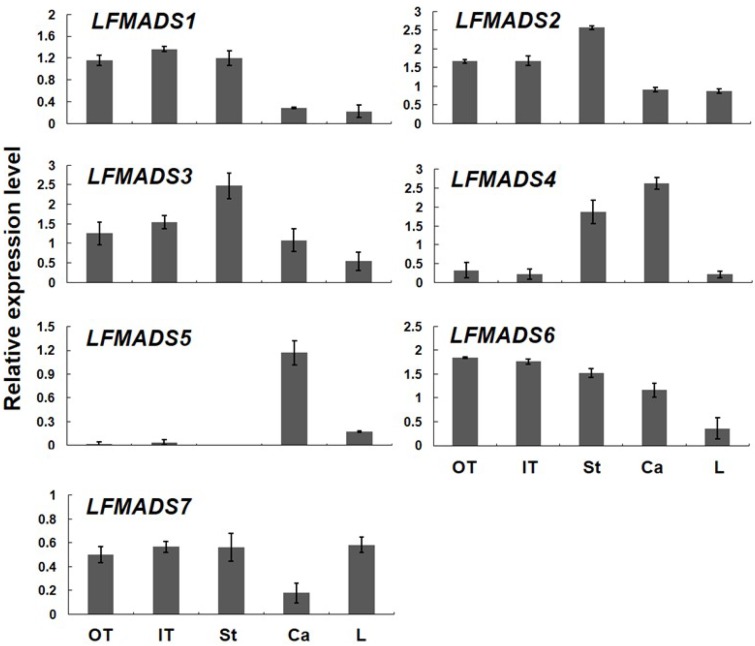
Quantitative analysis of seven LFMADSs gene expression in vegetative leaves and all four floral organs in *L. formosanum*. Total RNA was isolated from vegetative leaves and all four floral organs that were dissected from 2 cm flower buds in length, respectively. Real-time qRT-PCR analysis was performed for each collected sample and normalized with lily *GAPDH* (=1). Error bars indicate the standard deviation (*n* = 3). OT: outer tepal; IT: inner tepal; St: stamen; Ca: carpel; L: mature leaf. Primers used in qPCR reactions are listed in Table S6.

Many nongrass monocot flowers have two whorls of petaloid organs, which are called tepals. The floral organs of *L. formosanum* have three outer tepals, three inner tepals, six stamens and three carpels from outer whorl to inner whorl. We used real-time quantitative RT-PCR (qRT-PCR) to evaluate the gene expression in the four whorls, including outer tepals, inner tepals, stamans, and carpels by dissecting 2 cm floral buds in length, and mature leaves. Our data indicated that the transcripts of *LFMADS1*, *LFMADS2*, and *LFMADS3* were detected in outer tepal, inner tepal, and stamen, whereas relatively weaker signal in carpel and leaves (Figure 4). To explain this floral morphology, the modified ABC model was proposed [53]. This model was exemplified by the tulip, in which expansion and restriction of class B gene expression is linked to the transition of floral morphologies in whorl 1 [54]. The expression patterns of class B genes from many monocot species nicely fit into this model. Several class B genes were also isolated from other *Lilium* species. For example, one *DEF*-like (*LrDEF*) and two *GLO*-like (*LrGLOA* and *LrGLOB*) genes have been isolated from *L. regale* [55,56]. Northern blot analysis of the dissected floral organs showed that the *LRDEF* gene is expressed in outer and inner tepals and stamens. This expression pattern, like that of tulip, also supports the modified ABC model [54]. *LFMADS4* was highly expressed in stamen and carpel (Figure 4), similar expression pattern was also shown in other C-class genes such as *LLAG1* identified from *L. longiflorum* Thunb. [35], and *TAG1* and *TAGL1* identified from tomato [57]. The transcripts of *LFMADS5* were highly detected in carpel (Figure 4). Other D-class gene, *BdMADS2* for instance, identified from *Brachypodium distachyon* was also highly expressed in carpel [58]. In addition, the *LFMADS6* transcript showed major expression in all floral organs. Otherwise, the *LFMADS7* expression was detected at similar levels in all floral organs and mature leaves (Figure 4). Within E-class genes, such as *CastSEP3a/b/c* identified from *Crocus sativus L.* and *TaSEPs* from *T. aestivum L.* exhibited similar expression patterns in all floral organs [59,60]. Thus, our data are in agreement with theirs. In summary, our qRT-PCR results revealed that *LFMADS1-6* was dominantly expressed in reproductive organs compared to the vegetative leaves, while *LFMADS7* was expressed in reproductive as well as vegetative organs. These data are in accord with the results of our DEG analysis in that total reads of *LFMADS1-7* from transcriptome datasets showed similar total counts in PH-FB (1219) compared to PH-LF (1897) (Table 1).

### 2.5. Nuclear Localization of Seven lily MADS-Box Proteins

**Figure 5:**
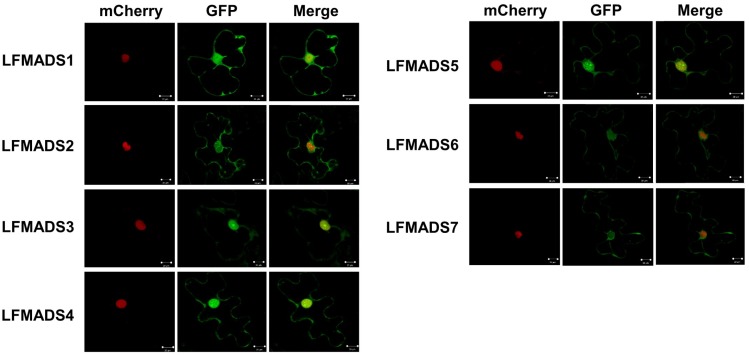
Subcellular localization of seven fluorescent LFMADS-mGFP proteins. Recombinant plasmids harboring a C-terminal mGFP fusion with LFMADSs were driven by the CaMV 35S promoter (35S::LFMADSs-mGFP). These seven recombinant proteins were transiently expressed in lily tepal by using the particle bombardment method. The NLS domain of VirD2 fused with mCherry in this study was used as the nuclear marker control. Overlay (merge) images are shown in the extreme right column. Bar = 20 μm.

The MADS-box proteins normally contain the conserved DNA-binding domain when function as transcription factors (TFs) and thus are localized in the nucleus. Most MADS–box proteins enclose nuclear localization signal sequences (K–K/R–x–K/R) in the N-terminal MADS domain, which are present in both partners for transporting the dimer into the nucleus [61]. Moreover, nuclear localization for MADS-box proteins isolated from other plant species, including *Arabidopsis*, rice, soybean, petunia, bamboo, and orchid have been validated experimentally [62,63,64]. Therefore, we also investigated the subcellular localization of these seven lily MADS-box proteins by fusing their C-terminus with mGFP and transiently expressed in the lily petal cells. The fluorescent signals of all seven LFMADSs-mGFP recombinant proteins were localized exclusively in the nucleus (Figure 5), suggesting their potential biological function as TFs.

### 2.6. Ectopic Expression of LFMADS2, LFMADS4, and LFMADS6 Cause Floral Defects in Transgenic Arabidopsis, with LFMADS4 and LFMADS6, Further Causing Early Flowering

**Figure 6:**
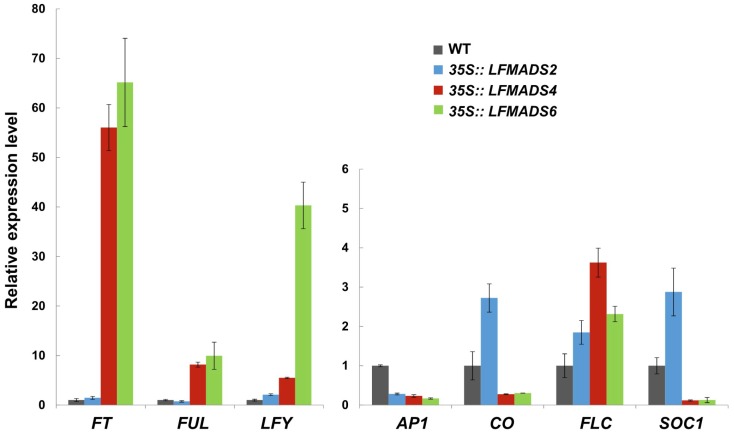
Detection of transcripts for endogenous flowering time genes and floral organ-identity genes in transgenic *Arabidopsis* overexpressing *LFMADS2*, *LFMADS4*, and *LFMADS6*, respectively. Relative transcription levels of endogenous flowering-related genes in *35S::LFMADS2*, *35S::LFMADS4*, and *35S::LFMADS6* transgenic *Arabidopsis* were determined by real-time qPCR analysis. Higher expression of *FT*, *FUL* and *LFY* transcripts were detected in *35S::LFMADS4* and *35S::LFMADS6* transgenic lines, while not observed in *35S::LFMADS2* transgenic *Arabidopsis* compared to the wild-type. All expression levels of genes were normalized against *TUB2* expression. Primers used in qPCR reactions are listed in Table S6.

To explore the functions of *LFMADSs* in floral organ identity and development, we selected and constructed recombinant plasmids harboring either the full-length of *LFMADS2* (representative B-class gene), *LFMADS4* (representative C-class gene), or *LFMADS6* (representative E-class gene) driven by the CaMV 35S promoter. These recombinant plasmids were transformed into *Arabidopsis* wild-type plants (Columbia ecotype) the same way as those performed by others [65]. Transgenic plants overexpressing *LFMADS4* and *LFMADS6* flowered earlier than the wild-type control, while this phenomenon was not found in *35S::LFMADS2* transgenic *Arabidopsis* (Table 2, Figure S10). Moreover, we performed qRT-PCR analysis to detect the expression of flowering-related genes involved in different flowering pathways and their relationship with overexpressing LFMADSs, leading to early-flowering phenotype in *35S::LFMADS4* and *35S::LFMADS6* transgenic plants. As a result, our data showed highly expressed floral integrator genes such as *FT*, *FUL*, and *LFY* that may have been involved in promoting flowering pathway. In accord with the flowering phenotype data, the notable change of the integrator genes expression was not detectable in *35S::LFMADS2* transgenic *Arabidopsis* (Figure 6).

**Figure 7:**
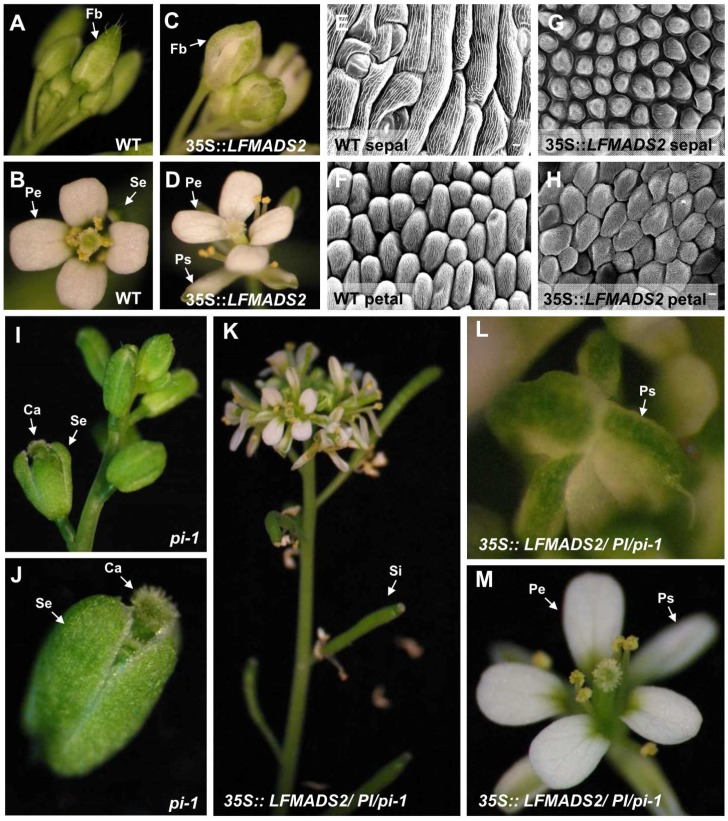
Phenotypic analysis of transgenic *Arabidopsis* overexpressing *LFMADS2* in wild-type (Columbia ecotype) and *pi-1* mutant (Landsberg *erecta*) background, respectively. (**A**,**B**) Normal floral bud and open flower phenotype in wild-type. Fb: floral bud. (**C**,**D**) Homeotic transformation of floral bud and open flower in *35S::LFMADS2* transgenic *Arabidopsis*. Of note, the flower showed the complete conversion of the sepal into white-like petaloid sepal (Ps) in the first whorl. (**E**,**F**) Scanning electron micrographs of surface cells of second whorl (petal) (**E**) and first whorl (sepal) cells (**F**) observed in the wild-type. (**G**,**H**) Scanning electron micrographs of surface cells of first whorl (petaloid sepal) (**G**) and second whorl (petal) cells (**H**) observed in the *35S::LFMADS2* transgenic *Arabidopsis*. The shape of cell and cell arrangement in the first whorl and second whorl showed phenotypically similar to the mature wild-type petal in (**E**). (**I**,**J**) The apical inflorescence structure (**I**) and flower phenotype (**J**) in homozygous *pi-1* mutant. The floral organ in *pi-1* lacks petal and stamen structures. (**K**,**M**) The phenotype of the apical inflorescence structure (**K**), floral bud (**L**), and open flower (**M**) in *35S::LFMADS2/PI/ pi-1* transgenic line C1 (*PI/pi* background). The flower in this transgenic line recovered four normal petals completely and sepals also showed a white margin similar to the petaloid sepal of (**D**). Bar = 20 μm. Se: sepal, Pe: petal; Ps: petaloid sepal; Si: siliques.

Furthermore, the phenotypic analysis of floral organs in *35S::LFMADS2* transgenic plants revealed the conversion of the sepals in the first whorl into petaloid-like sepal structures similar to ectopic expression of *PI* in *A. thaliana*. The sepals of outer whorl during normal flower development in wild-type (WT) were green (Figure 7A,B). However, the margin of petaloid sepals in *35S::LFMADS2* transgenic plants were pale-green and their buds were not completely enclosed in this transgenic plant (Figure 7C,D). The size of a petaloid sepal was similar to that of the petal, although the cells at the top and/or midrib remained the characteristics of sepals. Of note, our data (Figure 7C,D) are in agreement with other studies in which ectopic overexpression of other B-class MADS-box genes, such as *MdPI* and *ApGLO* led to the petaloid sepals change in transgenic *Arabidopsis* Columbia ecotype [66,67]. In addition, results of the arrangement of epidermal cells by scanning electron microscope (SEM) revealed that the cell morphology and arrangement of first whorls (petaloid sepals) in the transgenic plants are similar to the second whorls of petal cells in the WT (Figure 7E–H). To assess whether the function of LFMADS2 can restore the floral phenotype of the *pi-1* mutant which lacks of the petal and stamen structures in *Arabidopsis* (Figure 7I–J), we crossed *pi-1* with the pollen of one individual T_0_ transgenic plant in which *35S::LFMADS2* was introduced. The F_2_ transgenic C1 and C2 lines with *LFMADS2* had the recognition site for BsrI, thus the *35S::LFMADS2*/*PI*/*pi-1* transgenic lines were considered to have the *PI*/*pi* background (Figure S11). In addition, overexpressing LFMADS2 led to the sepal transformation into petaloid sepal in *35S::LFMADS2* with *PI/pi* background (Figure 7K–M). This transformation resulted in a phenotype similar to that of the WT in the presence of overexpressed LFMADS2 (Figure 7D), but not that of the original WT (Figure 7B). Our results, therefore, revealed that LFMADS2 may have the activity of B-class MADS-box transcription factor because the ectopic expression in *Arabidopsis* (*PI/pi* background) turned the sepals into petaloid organs, although restoration of petals and stamens formation was not observed in this heterozygote pi-1 mutant with the overexpressed LFMADS2. This notion also has been observed in previous studies on PI homologue of other plant species, in which ectopic overexpression of other B-class MADS-box genes *CabuPI* led to the petaloid sepals change in transgenic *Arabidopsis* with *PI*/*pi* background [68]. 

**Figure 8:**
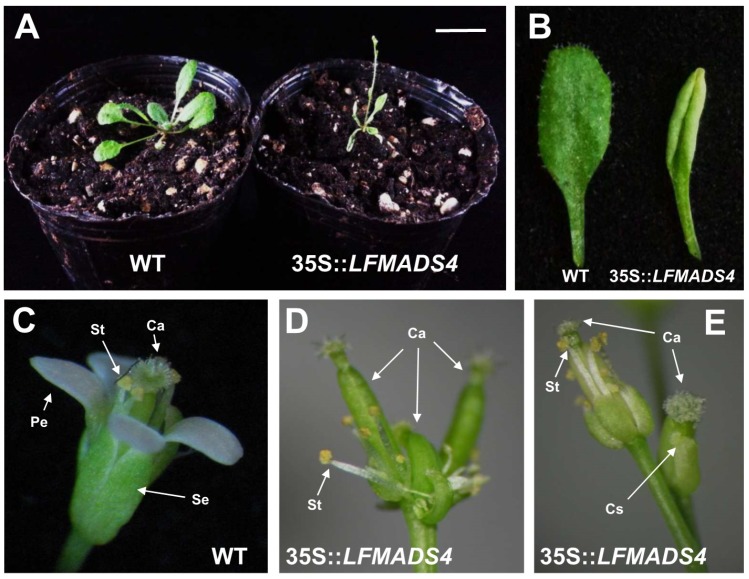
Overexpression of *LFMADS4* promotes flowering and causes curly rosette leaf and floral homeotic transformation in *Arabidopsis*. (**A**) Early-flowering phenotype in *35S::LFMADS4* transgenic *Arabidopsis* (right) compared to wild-type (WT) (left) under long-day condition. Bar = 1 cm. (**B**) Phenotype of curly rosette leaf in *35S::LFMADS4* transgenic *Arabidopsis* (right) compared to WT (left) with normal rosette leaf. (**C**) Normal floral structure in WT. (**D**,**E**) Floral structure shows homeotic transformation in the first whorl (sepal) and second whorl (petal) in *35S::LFMADS4* transgenic *Arabidopsis*. The flower with complete conversion of the sepal into carpel or carpellnoid sepal in the first whorl, and the petal into stamen in the second whorl are represented in the main inflorescence (**D**) and lateral inflorescence (**E**), respectively. Se: sepal, Pe: petal, St: stamen, Ca: carpel, Cs: carpellnoid sepal.

Next, we performed the phenotypic analysis for *35S::LFMADS4* transgenic *Arabidopsis* and discovered that floral transition plants showed extreme early flowering and produced fewer, smaller, and curly rosette leaves (Table 2, Figure 8A,B). In contrast to the inflorescence development in wild-type, fewer lateral inflorescence and 2,3 flowers were produced at the apical of the main inflorescence in *35S::LFMADS4* transgenic plants (Figure 8A,B). The normal floral organs in WT composed of four sepals in the first whorl and four petals in the second whorl (Figure 8C). The *35S::LFDMADS4* flowers bearing in main and lateral inflorescence exhibited as homeotic conversion of sepals into carpel-like structures (carpellnoid sepals) (Figure 8D,E). Stigmatic papillae and ovules were clearly observed in the first-whorl with carpel-like structures (Figure 8D,E). Notably, second-whorl organs (petals) were occasionally missing in these *35S::LFMADS4* flowers (Figure 8D,E). In *Arabidopsis*, ectopic expression of *AG* was shown previously to cause the homeotic conversion of sepals and petals into carpels and stamens, respectively [69,70]. Other reports also indicated that ectopic expression of C-class MADS-box genes resulted in similar phenotypes, including *LLAG1* from *L. longiflorum* Thunb. and *HpAG* from *Hosta plantaginea* [35,71].

**Figure 9:**
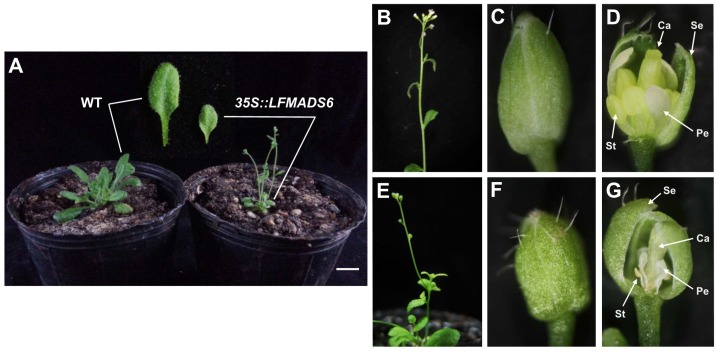
Overexpression of *LFMADS6* promotes flowering and causes different effects on inflorescence structure and floral defect in *Arabidopsis*. (**A**) Phenotype of leaf shape and floral transition in wild-type (WT) (left) and *35S::LFMADS6* transgenic *Arabidopsis* (right) under long-day condition. Bar = 1 cm. (**B**) Inflorescence structure of WT. (**C**) The morphology of floral bud in WT. (**D**) Dissection of a wild-type floral organ with normal developing sepal, petal, stamen and carpel. (**E**) Inflorescence structure of *35S::LFMADS6* transgenic *Arabidopsis*. (**F**) The morphology of a floral bud in *35S::LFMADS6* transgenic *Arabidopsis*. (**G**) Dissection of a *35S::LFMADS6* transgenic *Arabidopsis* floral bud revealed the aborted petal and stamen in the second and third whorl, respectively. Se: sepal, Pe: petal, St: stamen, Ca: carpel.

In addition, similar phenotypic analysis was performed for *35S::LFMADS6* transgenic *Arabidopsis*. These plants emerged as smaller rosette leaves and fewer, smaller, and more lateral inflorescence structures compared to WT (Figure 9A). The main inflorescence was developed normally in WT and its floral organ was composed of four petals in the second whorl and six stamens in the third whorl (Figure 9B–D). Unlike WT, the branch inflorescences were developed, and the floral structure exhibited aborted petals and stamens in *35S::LFMADS6* transgenic plants (Figure 9E–G). Previous studies indicated that the ectopic expression of functional E-class genes of MADS-box family caused different effects on plant development in transgenic lines, relating to the functional divergence of E-class MADS-box genes that may play various roles in regulating the floral organ identity in all four floral organs. For example, overexpression of an E-class homologous gene, *Wheat SEPALLATA* (*WSEP*), caused transgenic plants with four to five smaller curly leaves, early flowering, and produced terminal flowers. However, no morphological changes in floral organs were observed [72]. In our studies, ectopic expression of *LFMADS2*, *LFMADS4*, and *LFMADS6* in *Arabidopsis* caused floral defects, which was not due to the co-suppression of *Arabidopsis* floral organ-identity genes. Our qRT-PCR results revealed that the transcription levels of endogenous floral organ-identity genes (*AP1*, *AP2*, *AP3*, *PI*, *SEP1*, *SEP2*, *SEP3*, and *SEP4*) showed similar pattern with that observed in WT flower (Figure S12). 

### 2.7. Overexpression of the LFMADS2, LFMADS4, and LFMADS6 Genes Induced Early Flowering in Transgenic Tobacco

**Figure 10:**
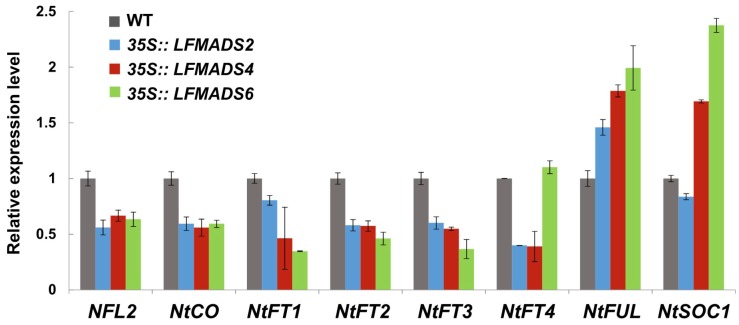
Effect of the overexpressed *LFMADS2*, *LFMADS4*, and *LFMADS6* on the transcript levels of regulatory genes related to the flowering time in tobacco. Relative transcript levels of *NFL2*, *NtCO*, *NtFT1*, *NtFT2*, *NtFT3*, *NtFT4*, *NtFUL*, and *NtSOC1* were determined by real-time qPCR analyses of WT and transgenic tobaccos, including *35S::LFMADS2*, *35S::LFMADS4*, and *35S::LFMADS6* seedlings. Each bar represents the average of three replicates and the standard deviation is shown. All the expression levels of each gene were normalized against *NteIF4A10* expression. Primers used in qPCR reactions are listed in Table S6.

**Figure 11:**
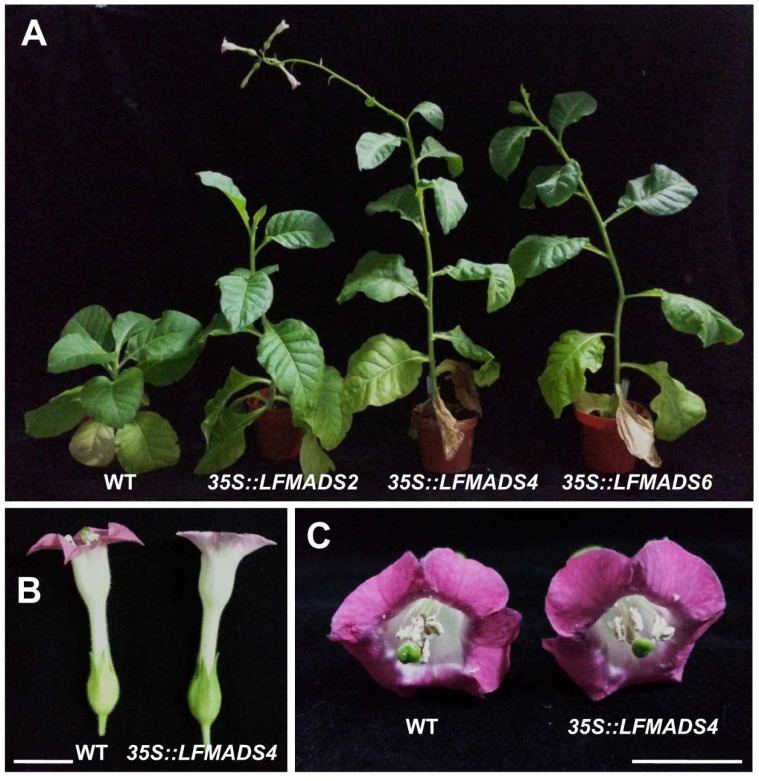
Phenotypic analysis of *35S::LFMADS2*, *35S::LFMADS4*, and *35S::LFMADS6* transgenic tobaccos. (**A**) Overexpression of *LFMADS2*, *LFMADS4*, and *LFMADS6* promotes flowering in tobaccos under the long-day condition. (**B**,**C**) Phenotypic analysis of floral organs of WT and *35S::LFMADS4* transgenic tobaccos. No obvious difference in morphology, shape, arrangement, and color is shown in terms of floral organ between WT and transgenic tobacco. Bar = 1 cm.

To analyze the biological roles of *LFMADSs* in floral transition and determine the floral organ-identity in other plant species, we also ectopically expressed *LFMADS2*, *LFMADS4*, and *LFMADS6* under the control of CaMV 35S promoter in tobacco. We found that *35S::LFMADS2*, *35S::LFMADS4*, and *35S::LFMADS6* transgenic tobaccos showed significantly early flowering compared to WT (Table 3). Similar results also revealed in ectopically expressed *35S::LFMADS4* and *35S::LFMADS6* in transgenic *Arabidopsis* plants (Figure 8A and Figure 9A). In addition, the effect of the overexpressed *LFMADS2*, *LFMADS4*, and *LFMADS6* on the transcript levels of regulatory genes related to the flowering time in tobacco was examined. Relative transcript levels of the flowering-related genes, including *NFL2*, *NtCO*, *NtFT1*, *NtFT2*, *NtFT3*, *NtFT4*, *NtFUL I*, and *NtSOC1* [73,74,75] were determined by real-time qPCR analyses of WT and transgenic tobaccos. Our real-time qRT-PCR analysis indicated that *LFMADS2*, *LFMADS4*, and *LFMADS6* significantly expressed integrator genes, *NtFUL* and *NtSOC1*, which may affect floral transition causing early flowering in transgenic tobaccos (Figure 10 and Figure 11A). The morphology of floral organs in terms of shape, arrangement, and color was indistinguishable between *35S::LFMADS4* transgenic tobacco and WT (Figure 11B,C). Similar results were also observed for *35S::LFMADS2* and *35S::LFMADS6* transgenic tabaccos. Previous studies have shown that constitutively expressed *GmMADS28*, identified from soybean, in transgenic tobacco resulted in early flowering, effects on floral organ numbers, petal identity, and sterility [76]. *PhPI15*, identified from *Phalaenopsis* orchid, when ectopically expressing in tobacco showed male-sterile phenotype [77]. Overexpressed sugar beet *BvM14-MADS* box gene in transgenic tobacco exhibited increased length of gynoecium in floral organ, low seed weight, and slightly delayed flowering time. In addition, the corolla of the transgenic plants was usually smaller than that of the WT, and the color was white compared to pink in the WT [78]. These data, therefore, indicate the function of these LFMADS proteins may not be involved in floral organ identity in tobacco.

### 2.8. LFMADSs form Protein Complex with Various MADS-Box Proteins

**Figure 12:**
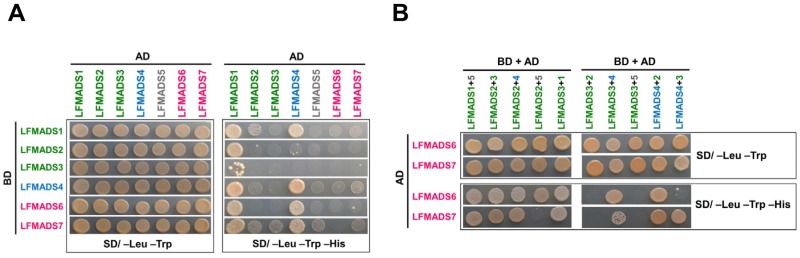
Analysis of protein-protein interactions among B-, C-, D-, and E-class LFMADS proteins by using the GAL4 yeast two-hybrid system. (**A**) Co-transformed yeast cells with two constructs, including GAL4AD-LFMADSs (AD, on the top) and GAL4BD-LFMADSs (BD, on the left). Each transformant was diluted to 10^6^ cells and spotted on control medium (SD/–Leu–Trp) and selective medium (SD/–His–Leu–Trp), respectively. (**B**) Co-transformed yeast cells with three constructs, including BD + AD from (**A**) (on the top), and then transformed with another GAL4AD-LFMADSs construct (AD, on the left). Each transformant was diluted to 10^6^ cells and spotted on control medium (SD/–Leu–Trp) and selective medium (SD/–His–Leu–Trp), respectively. All yeast cells grew on control medium indicating correct co-transformations. The B-, C-, D-, and E-class LFMADSs are labeled in green, blue, grey, and pink, respectively.

According to the genetics and yeast two- and three-hybrid studies, the MADS-box proteins were able to form multimeric complexes with other proteins and a quartet model was further hypothesized, forming tetrameric complexes [26,79]. To further understand whether these LFMADS multimeric complexes would have effects on the floral organ identity, we investigated the protein interaction patterns of the LFMADSs by using the yeast two-hybrid analysis. Our data indicated that the transformed yeast cells co-expressing the GAL4AD-LFMADS1 fusion protein with different GAL4BD-LFMADS1 to 7 fusion proteins formed a strong LFMADS1 homodimer and heterodimer (except D-class LFMADS5), while LFMADS2 and LFMADS3 preferred to form heterodimer with LFMADS1. They did not form homodimer with themselves even though they both were classified as B-class MADS-box proteins (Table 4, Figure 12A). The C-class GAL4AD-LFMADS4 together with other respective GAL4BD-LFMADSs (except B-class LFMADS2 and LFMADS3), grew robustly on the selective medium lacking Leucine, Tryptophan, and Histidine (SD/–His–Leu–Trp) (Figure 12A). In addition, yeast cells co-transformed with the D-class GAL4AD-LFMADS5 and either the B-class (GAL4BD-LFMADS2 or GAL4BD-LFMADS3) or the E-class GAL4BD-LFMADS7 grew weakly on the selective SD/–His–Leu–Trp medium (Table 4, Figure 12A). Previous studies revealed that the E-class MADS protein SEP3 plays an important role as a glue to mediate multimerization within MADS proteins in *Arabidopsis* and *orchid* [80,81]. Our assays showed E-class LFMADS proteins (LFMADS6 and LFMADS7) interacted weakly with other LFMADSs proteins, possibly due to fact that the binding abilities of heterodimers are unstable compared to trimeric protein complexes bridged by SEP proteins. We thus tested the hypothesis that whether the LFMADS6 and LFMADS7 proteins can function as a glue to mediate and strengthen the interactions among LFMADS proteins by co-transforming three constructs into the yeast cells. Our data revealed that B-class LFMADS1 belonging to AP3 lineage while LFMADS2 belonging to PI lineage. However, they both interacted with the D-class LFMADS5 and E-class LFMADSs to form trimeric complexes. By contrast, another B-class, LFMADS3 classified into PI lineage, did not interact with the D-class LFMADS5 forming multimeric complexes even in the presence of E-class LFMADSs (Table 5, Figure 12B). The weak binding signals among E-class LFMADSs (LFMADS6 and LFMADS7), B-class LFMADSs (LFMADS2 and LFMADS3), and D-class LFMADS5 in two-hybrid analysis were greatly increased in the three-hybrid analysis (Table 5, Figure 12B). The studies of these protein–protein interactions, therefore, suggested that E-class LFMADSs may form multimeric complexes with other B-, C-, and D-class LFMADSs to determine the floral organ identity in Formosa lily.

## 3. Materials and Methods 

### 3.1. Plant Materials and Growth Conditions

Formosa lily (*L. formosanum*) used in this study was grown in the field in the Hualien District Agricultural Research and Extension Station, Council of Agriculture, Executive Yuan, Hualien, Taiwan. Experiments were performed using *Arabidopsis* plants of Columbia ecotype as the wild-type (WT). Sterilized seeds for *Arabidopsis* were incubated in water at 4 °C for 2 days, followed by placed on 1/2 × Murashige and Skoog (MS) medium [82]. After cold treatment, the seedlings were grown in growth chambers under long-day conditions (16 h light/8 h dark) at 22 °C for 10 days before being transplanted into soil. The light intensity of the growth chambers was 150 μE m^−2^s^−1^.

### 3.2. RNA Isolation, Illumina Sequencing, Sequence Annotation, and Differential Gene Expression (DGE) Analysis

The total RNA from the two tissues (mix 0.5–3 cm floral buds in length and mature leaf) of Formosa lily was extracted using the TRIzol method (Invitrogen, Carlsbad, CA, USA) according to the manufacturer’s protocols. Purified RNA was quantified using Nanodrop (Thermo Scientific, USA) and agarose gel electrophoresis was performed to check the quality of RNA. The sequencing library was prepared using 5 μg of RNA from each sample (PH-FB for mix floral buds or PH-LF for mature leaves) and sequenced by using the Illumina HiSeq^TM^ 2000 high throughput sequencing platform with paired-end technology following the manufacturer’s instructions. After removing the adaptor sequences, empty reads, and low-quality sequences, a large number of high-quality reads were obtained, and the resultant datasets are available in the NCBI Sequence Read Archive (SRA) under the accession numbers SRX3822957 and SRX3822958 for PH-FB and PH-LF transcriptome datasets, respectively. After combining the high-quality clean reads from PH-FB and PH-LF transcriptome datasets, the de novo assembly was performed using the Trinity *de novo* program to generate the ALL transcriptome datasets [83]. The functions of the assembled transcripts (contigs and unigenes) were first aligned by BlastX to the protein databases of NR, Swiss-Port, KEGG, and COG specifying the E-values of less than 10^−5^. According to the annotations obtained from Nr, we used the Blast2GO program to further gain the GO elucidations for our unigenes [40]. Thereafter, the WEGO software [26] was used to run GO functional classifications for all unigenes and to understand the distribution of the functions of all of the genes in Formosa lily at the macro level. The unigene sequences were also aligned to the COG database to predict and classify possible functions. Furthermore, KEGG Pathway assignments were performed according to KEGG pathway database and related software applications (available online: https://www.genome.jp/kegg/). Differential gene expression (DGE) analysis was used to compare the differences in gene expression between developing floral buds (PH-FB transcriptome datasets) and mature leaves (PH-LF transcriptome datasets). All clean tags were mapped to the reference sequences, and DGE counts were normalized using the RPKM (Reads Per kb per Million reads) method [84,85]. The RPKM represents the expression level of a given unigenes sequence. We finally identified differentially expressed genes between these two samples by FDR (False Discovery Rate) method to determine the appropriate threshold of the *p* value in multiple tests and analysis. In our study, we used FDR ≤ 0.001 and a fold change (the ratio of expression between two groups) ≥ 2 as the criteria to judge the significance of gene expression difference and screen the differentially expressed genes (DEGs). Hierarchical cluster analysis was used to show the expression patterns of genes with the same or similar expression behavior, and these DEGs were also annotated against GO and KEGG databases for functional enrichment analysis. The expression level of these selected DEGs expression was confirmed by qRT-PCR. 

### 3.3. Cloning of the cDNA for LFMADS Genes from the Formosa Lily

To validate the transcriptome DGE data, seven *LFMADS* genes were chosen and full-length genes were cloned for the following gene expression and functional analysis. Total RNAs were isolated from a mixture of 0.5, 1, and 2 cm long floral buds of Taiwan lily using TRIzol reagent (Invitrogen, Carlsbad, CA, USA) according to the manufactures’ instructions. First-strand cDNA synthesis was prepared using an oligo(dT) primer and 2 μg of RNA in conjunction with a ImProm-II^TM^ reverse transcription system (Promega, Madison, WI, USA). PCR amplification was carried out by using *LFMADS* gene-specific primer sets (Table S6). Amplified cDNAs were cloned into pGEM-T easy vector (Promega, Madison, WI, USA) and further sequenced to check the specificity of the amplification products. 

Similar strategy was used to generate transgenic tobacco plants (*N. tabacum* L.) in order to overexpress *LFMADSs* by using the *Agrobacterium*-mediated transformation as described previously [86]. These tobacco transformants were selected on TSM medium (MS medium supplemented with 3% sucrose, 0.1 mg/L 1-Naphthylacetic acid (NAA), 1 mg/L N6-Benzyladenine (BA) and 8 g/L agar) containing 50 μg/mL hygomycin. Shoots would grow from the edge of explants and separated from the explants after 3–5 weeks. We then cut the shoots off and transferred them into TRM medium (MS medium supplemented with 3% sucrose, 0.1 mg/L 1-Naphthylacetic acid (NAA) and 8 g/L agar) with the same antibiotics. Once rooting, tobacco seedlings were transferred and grown in pots containing soil in a growth room at 26 °C under long-day (16 h light/8 h dark) conditions.

### 3.4. Sequence Alignment and Phylogenetic Analyses

The deduced amino-acid sequences of LFMADS1 to 7 were aligned with the published B-, C-, D- and E-class MADS-like protein sequences from other plant species, obtained from the database at NCBI server (available online: http://www.ncbi.nlm.nih.gov/). Subsequently, the calculation of bootstrap values was conducted using the ClustalX version 1.83 (European Bioinformatics Institute, Hinxton, Cambridge, UK) [87,88]. The genetic distances were calculated by the Kimura 2-parameter [89], and the phylogenetic tree was generated with MEGA6 by the neighbor-joining (N.J.) method [90]. Numbers on the tree nodes are the bootstrap values from the 1000 replicates. 

### 3.5. RT-PCR and Real-Time Quantitative RT-PCR (qRT-PCR)

Total RNA (2 µg), extracted from the various organs, including outer tepals (OT), inner tepals (IT), stamens (St), carpel (Ca), and mature leaves (L) of Taiwan lily; or from the leaves of 35S::*LFMADSs* transgenic *Arabidopsis*; or from the tobacco plants, was used for cDNA synthesis by reverse transcription of a 15 µL reaction mixture using the ImProm-II^TM^ reverse transcription system (Promega, Madison, WI, USA) according to the manufacture’s protocols. One microliter of cDNA sample from RT reaction was further used for 30 cycles of a PCR reaction as follows: the denaturing step was 94 °C for 30 s, the annealing step was 58 °C for 45 s, and the extension step at 72 °C for 90 s. The final elongation was performed at 72 °C for 7 min. The resulting PCR products in each reaction was separated and analyzed by electrophoresis in 1.5% (*w*/*v*) agarose gels. Quantitative RT-PCR (qRT-PCR) was performed with gene-specific primers, using the *TUB2* primer set as an internal control. The relative expression of the three genes was normalized to the expression level of *TUB2* in *Arabidopsis* or in tobacco with biological repeats in triplicates. The qRT-PCR was performed on a Chromo4 Continuous Fluorescence Detector (Bio-Rad, Hercules, CA, USA) by using the KAPA SYBR FAST Universal qPCR Kit (KAPA BIOSYSTEMS) as instructed by the manual’s recommendations. The comparative Ct method was finally used to determine the relative gene expression level. These results were analyzed with BioRad CFX Manager (Bio-Rad). Three qPCR replicates were performed for each sample. All primers used in this study are listed in Table S6.

### 3.6. Genomic DNA-PCR and Genotype Analysis

To assess whether the function of LFMADS2 can restore the floral phenotype of the pi-1 mutant which lacks of the petal and stamen structures in *Arabidopsis* (Figure 7I,J), we crossed pi-1 with the pollen of one individual T_0_ transgenic plant in which 35S::LFMADS2 was introduced. The introduction of LFMADS2 into some F2 transgenic lines (35S::LFMADS2/pi-1) were selected on MS medium with antibiotic and confirmed the presence of LFMADS2 fragment by genomic DNA-PCR. The genomic DNA was isolated from the rosette leaves of these F2 plants by using a commercially available kit (DNeasy Plant Mini Kit, Qiagen, Valencia, CA, USA) following the manufacturer’s instructions. The partial fragments of the PI (WT) and mutant pi were amplified from the same genomic DNA of aforementioned transgenic plants by PCR with pi-1/F and pi-1/R primer sets (Table S6). The PCR conditions were 94 °C for 1 min, 50 °C for 1 min, and 72 °C for 2 min for 28 cycles. Of note, the PCR products of pi-1 mutant (pi/pi background) could be digested with the restriction enzyme BsrI (NEW ENGLAND Bio Labs, UK), producing 530-bp and 570-bp fragments, while not the WT (PI/PI background) [66] (Figure S11).

### 3.7. Subcellular Localization of Green Fluorescent LFMADSs Fusion Proteins 

Full-length coding region for *LFMADSs* were amplified by PCR with gene-specific 5’ and 3’ primers, containing the generated Xba I and Kpn I recognition sites (Table S6) to facilitate cloning of the respective cDNAs. Amplified PCR fragments were ligated into the constitutive expression vector pEpyon-12K, resulting in LFMADSs-mGFP fusion proteins expressed under the control of the CaMV35S promoter. These reporter constructs were isolated and transformed into lily petal cells using bombardment transformation method [91]. Fluorescence in the transformed cells was observed on a Zeiss LSM 510 META laser-scanning confocal microscope using an LD C-Apochromat 409/1.1 W objective lens [91].

### 3.8. Binary Vector Construction, Plant Transformation, and Analysis of Transgenic Plants 

The full-length cDNA encoding LFMADSs were each generated by PCR using gene-specific primer sets and further cloned into the binary vector pCambia1390 under the control of the CaMV35S promoter. The orientation of the sense constructs was determined by PCR analysis. These sense constructs were then transformed into *Arabidopsis* plants using the floral dip method [92] and the *Agrobacterium tumefaciens* GV3101 strain was used for this transformation. Transformed seedlings (T_1_ seedlings) were selected and further verified by genomic DNA PCR and RT-PCR analyses if they survived in the 1/2 MS medium containing 50 μg/mL hygomycin. The self-pollinated T_2_ were also grown in the identical conditions.

### 3.9. Scanning Electron Microscopy

For scanning electron microscope (SEM) analysis, the flowers of transgenic *35S::LFMADSs* containing *Arabidopsis* were fixed in two steps: first by using a mixture of 2% paraformaldehyde and 2.5% glutaraldehyde in 0.1 M buffer (pH 7.4) for 1 h, followed by 1% OsO_4_ in 0.1 M cacodylate buffer (pH 7.4) for 1 h. After dehydration in a graded ethanol series, the dehydrated flowers were dried for 3 h (Hitachi critical point dryer, HCP-2; Hitachi Koki), sputter-coated with gold in 180 s bursts (Hitachi ion sputter, E-1010; Hitachi Koki), and the specimens were examined and photographed under a scanning electron microscope (Hitachi S-4700) with an accelerating voltage of 15 kV.

### 3.10. Yeast-Two Hybrid Analysis and Spotting Assay

The full-length cDNA for all the *LFMADSs* genes were each generated by PCR using the gene-specific primer sets. The PCR fragments were then ligated into the plasmids pGBKT7 (binding domain vector, bait construct) or pGADT7 (activation domain vector, prey construct). Subsequently, yeast two-hybrid analyses were carried out according to the Matchmaker Two-Hybrid System II User Mannual (Clontech, Palo Alto, CA, USA). Specific bait and prey constructs were transformed into yeast strain AH109 simultaneously. Protein interactions were determined by the growth conditions on the selective synthetic defined medium lacking tryptophane, leucine, and histidine (SD/–Trp–Leu–His) as well as on the control SD/–Leu–Trp) medium. Trimeric protein-protein interactions were analyzed as described by Pan et al. [83]. Briefly, the yeast strains were constructed by co-transformation of three constructs, including two pGADT7 constructs and one pGBKT7 construct, each containing respective *LFMADSs* genes. Protein–protein interactions were finally determined by spotting assays. All the spotting assays were at least repeated three times.

## 4. Conclusions

In this study, we assembled *L. formosanum* transcriptome datasets and performed DGE analysis from two different tissues, including developing floral buds and mature leaves. To validate our assembled transcriptome datasets, we selected and isolated seven full-length *LFMADS* genes, homologus to B-, C-, D-, and E-class floral organ identity genes for further analysis of their potential biological functions. Among these genes, *LFMADS1-6* was differentially upregulated, while *LFMADS7* with similar expression pattern when data from the developing floral buds were compared to those of the mature leaves, therefore, *LFMADS7* was used as a control in some of our assays. Our results revealed that the expression patterns of these *LFMADS1-7* genes in 2-cm floral organs or mature leaves correlate well with the expression profiles from our DGE analysis, indicating the reliability of our assays. According to the phylogenetic analysis and subcellular location results of these LFMADSs proteins, *LFMADS* genes belong to the MADS-box containing transcription factor gene family, involved in regulating the flower development in Formosa lily. In addition, functional analysis showed that *LFMADS2*, *LFMADS4*, and *LFMADS6* effect on floral organ identity in transgenic *Arabidopsis*. These LFMADSs were also shown to be potentially involved in the floral transition in transgenic *Arabidopsis* and tobacco as a result of the upregulated floral integrator genes such as *FT*, *SOC1*, *FUL*, and *LFY*. Furthermore, we provided the evidence supporting the biological function of E-class LFMADSs: LFMADS6 and LFMADS7 as a bridge to form multimeric complexes with other B-, C-, and D-class LFMADSs in determining the floral organ identity in Formosa lily. Taken together, the characterization of *L. formosanum* transcriptome data provides an effective tool and sequences resource for better understanding the molecular mechanisms of cellular processes, including development of mature leaves and flowers, as well as application for future molecular breeding in *Lilium* species. 

## Figures and Tables

**Table 1:** Characteristics of seven genes encoding MADS-box proteins identified in *L. formosanum*.

Gene Name	ALL Transcriptome Unigene ID	PH-FB Total Counts	PH-LF Total Counts	Gene Length (bp)	Number of Amino Acids	MW (kDa) ^1^	Isoelectric Point ^1^	Accession Number
*LFMADS1*	FL_194	8071	13	687	228	26.18	8.68	AHY82568
*LFMADS2*	FL_2387	6698	7	633	210	24.53	8.99	AHY82569
*LFMADS3*	FL_4062	2144	0	546	181	20.98	9.14	AHY82570
*LFMADS4*	FL_3393	1067	44	738	245	28.62	9.21	AHY82571
*LFMADS5*	FL_12403	291	0	699	232	26.72	9.03	AHY82572
*LFMADS6*	FL_663	15291	1	729	242	27.58	9.16	AHY82573
*LFMADS7*	FL_1675	1219	1897	741	246	28.17	7.05	AHY82574

^1^ Detailed LFMADSs protein characteristics were predicted by ExPASy online service (http://web.expasy.org/compute_pi/).

**Table 2:** Bolting time of the transgenic *35S::LFMADS2*, *35S::LFMADS4*, and *35S::LFMADS6* plants compared to wild-type (Columbia ecotype).

Plant Genotype ^1^	Number of Plants (n)	Days to Flowering ^2^
*35S::LFMADS2*	45	28.8 ± 0.8
*35S::LFMADS4*	45	19.8 ± 0.8
*35S::LFMADS6*	45	24.0 ± 0.7
Columbia wild-type	45	29.2 ± 1.3

^1^ The seedlings were grown in growth chambers under long-day condition (16 h light/8 h dark) at 22 °C for 12 days before transplanted to soil. ^2^ Days from sowing to emergence of the main inflorescence (1 cm) (±SD).

**Table 3:** Flowering time of the *35S::LFMADS2*, *35S::LFMADS4*, and *35S::LFMADS6* transgenic tobacco plants under long-day (16 h light/8 h dark) conditions.

Transgenic Tobacco Line	Number of Plants (*n*)	Flowering Time (days)
WT	30	81.8 ± 0.8
*35S::LFMADS2*	30	45.2 ± 0.7
*35S::LFMADS4*	30	39.6 ± 0.5
*35S::LFMADS6*	30	41.6 ± 0.5

**Table 4:** The relationships of protein–protein interaction among B-, C-, D-, and E-class LFMADS proteins analyzed by GAL4 yeast two-hybrid system.

	B-Class			C-Class	D-Class	E-Class	
	LFMADS1 ^1^	LFMADS2	LFMADS3	LFMADS4	LFMADS5	LFMADS6	LFMADS7
LFMADS1 ^2^	+++++ ^3^	++	–	++++	–	+	+
LFMADS2	+++++	–	–	–	–	–	–
LFMADS3	+++	–	–	–	–	–	–
LFMADS4	+++++	–	–	+++++	–	–	+
LFMADS5	n.d. ^4^	n.d.	n.d.	n.d.	n.d.	n.d.	n.d.
LFMADS6	+++++	–	–	++++	–	–	–
LFMADS7	+++++	+	+	++++	+	–	+

^1^ GAL4BD-LFMADSs constructs. ^2^ GAL4AD-LFMADSs constructs. ^3^ Interactions between transformants are scored as “+++++”, “++++”, “+++”, “++”, “+” and “–“, indicating very strong, strong, weak and negative, respectively, on SD/–His–Leu–Trp selective medium. ^4^ n.d. means no detection in this study.

**Table 5:** Analysis of protein–protein interactions between the E-class LFMADSs (LFMADS6 and 7) and other B-, C-, D- class LFMADS proteins performed by GAL4 yeast two-hybrid system.

	LFMADS 1 ^1^+5 ^2^	LFMADS 2+3	LFMADS 2+4	LFMADS 2+5	LFMADS 3+1
LFMADS6 ^3^	+++++ ^4^	++++	++++	+++++	+++++
LFMADS7	+++++	+++++	+++++	–	+++++
	**LFMADS** **3+2**	**LFMADS** **3+4**	**LFMADS** **3+5**	**LFMADS** **4+2**	**LFMADS** **4+3**
LFMADS6	–	+++++	–	+++++	–
LFMADS7	–	++	–	+++++	+++++

^1^ GAL4BD-LFMADSs constructs. ^2^ GAL4AD-LFMADSs constructs. ^3^ Yeast cells are transformed with another GAL4AD-LFMADSs constructs. ^4^ Interactions for transformants are scored “+++++,” “++++,” “+++,” “++,” “+,” and “–,” indicating very strong, strong, weak, and negative, respectively, on SD/–His–Leu–Trp selective medium.

## Supplementary Materials

Supplementary materials can be found at http://www.mdpi.com/1422-0067/19/8/2217/s1.

## Abbreviations

DGE	differential gene expression
TF	transcription factor
NCBI	National Center for Biotechnology Information
SRA	Sequence Read Archive
FDR	False Discovery Rate
DEGs	differential expressed genes
SEM	scanning electron microscope
