# Supplementary material for: Overexpression of Lilium formosanum MADS-box (LFMADS) Causing Floral Defects While Promoting Flowering in Arabidopsis thaliana, Whereas Only Affecting Floral Transition Time in Nicotiana tabacum
Source: Int J Mol Sci. 2018 Jul 29;19(8):2217. doi: 10.3390/ijms19082217 (PMC6121541; doi:10.3390/ijms19082217)
Supplement: Supplementary file 1 [file ijms-19-02217-s001.zip › ijms-329608 suppl/Supplementary material-IJMS329608 07172018.pdf]

International Journal of
Molecular Sciences

MDPI

Supplementary Materials: Overexpression of *Lilium formosanum MADS-box* (*LFMADS*) genes causing floral defects while promoting flowering in *Arabidopsis thaliana*, wherease only affecting floral transition in *Nicotiana tabacum*

Wan-Yu Liao, Lee-Fong Lin, Ming-Der Lin, Sheng-Che Hsieh, Althea Yi-Shan Li, Yueh-Shiah Tsay and Ming-Lun Chou

Table S1. Output statistics of sequencing.

Samples	Total clean reads [1]	Total clean nucleotides (nt) [1]	Q20 percentage [2]	N percentage [3]	GC percentage [4]
PH-FB	41,207,408	6,181,111,200	97.46%	0.00%	51.30%
PH-LF	42,376,902	6,356,535,300	96.97%	0.00%	51.93%

[1] Total reads and total nucleotides are actually clean reads and clean nucleotides. The total nucleotides should be greater than contract provision. [2] The Q20 percentage is the proportion of nucleotides with a quality value higher than 20. [3] The N percentage is the proportion of unknown nucleotides in clean reads.[4] The GC percentage is the proportion of guanidine (G) and cytosine (C) nucleotides among total nucleotides.

Table S2. Statistics of the assembly quality for L. *formosanum* transcriptome dataset.

Classification	Sample	Total number	Min length (nt) [1]	Max length (nt) [2]	Mean length (nt) [3]	N50 [4]	N90 [4]	GC percentage [5]
Contig	ALL	18,041	200	9,594	1109.3	1,548	558	46.7%
Unigene	ALL	23,807	200	9,772	866.3	1,366	360	47.4%
Contig + Unigene	ALL	41,848	200	9,772	971.03	1,456	436	47.06%

[1] The minimal length for all unigenes. [2] The maximal length for all unigenes. [3] The mean length for all unigenes. [4] N50 or N90: length of the smallest transcripts in the total added up contigs' length that is longer than the 50% (or 90%) of the total contigs' length. [5] shows the percentage of GC.

Table S3. Categorization of Formosa lily unigenes into KEGG biochemical pathways.

Number	KEGG Pathway	ALL unigenes with pathway annotation (24,306)	Pathway ID	Level 1	Level 2
1	Metabolic pathways	4129 (16.99%)	ko01100	Metabolism	Global and overview maps
2	Biosynthesis of secondary metabolites	2124 (8.74%)	ko01110	Metabolism	Global and overview maps
3	Plant-pathogen interaction	634 (2.61%)	ko04626	Organismal Systems	Environmental adaptation
4	Ribosome	625 (2.57%)	ko03010	Genetic Information Processing	Translation

5	RNA transport	620 (2.55%)	ko03013	Genetic Information Processing	Translation
6	Spliceosome	597 (2.46%)	ko03040	Genetic Information Processing	Transcription
7	Protein processing in endoplasmic reticulum	576 (2.37%)	ko04141	Genetic Information Processing	Folding, sorting and degradation
8	Endocytosis	544 (2.24%)	ko04144	Cellular Processes	Transport and catabolism
9	Starch and sucrose metabolism	514 (2.11%)	ko00500	Metabolism	Carbohydrate metabolism
10	Plant hormone signal transduction	498 (2.05%)	ko04075	Environmental Information Processing	Signal transduction
11	Carbon metabolism	482 (1.98%)	ko01200	Metabolism	Global and overview maps
12	Purine metabolism	476 (1.96%)	ko00230	Metabolism	Nucleotide metabolism
13	Pyrimidine metabolism	457 (1.88%)	ko00240	Metabolism	Nucleotide metabolism
14	mRNA surveillance pathway	428 (1.76%)	ko03015	Genetic Information Processing	Translation
15	Biosynthesis of amino acids	422 (1.74%)	ko01230	Metabolism	Global and overview maps
16	Phenylpropanoid biosynthesis	383 (1.58%)	ko00940	Metabolism	Biosynthesis of other secondary metabolites
17	Ubiquitin mediated proteolysis	343 (1.41%)	ko04120	Genetic Information Processing	Folding, sorting and degradation
18	RNA degradation	269 (1.11%)	ko03018	Genetic Information Processing	Folding, sorting and degradation
19	RNA polymerase	262 (1.08%)	ko03020	Genetic Information Processing	Transcription
20	Oxidative phosphorylation	262 (1.08%)	ko00190	Metabolism	Energy metabolism
21	Amino sugar and nucleotide sugar metabolism	237 (0.98%)	ko00520	Metabolism	Carbohydrate metabolism
22	Glycolysis / Gluconeogenesis	224 (0.92%)	ko00010	Metabolism	Carbohydrate metabolism
23	Pentose and glucuronate interconversions	199 (0.82%)	ko00040	Metabolism	Carbohydrate metabolism
24	Glycerophospholipid metabolism	196 (0.81%)	ko00564	Metabolism	Lipid metabolism
25	Ribosome biogenesis in eukaryotes	194 (0.8%)	ko03008	Genetic Information Processing	Translation
26	Circadian rhythm - plant	190 (0.78%)	ko04712	Organismal Systems	Environmental adaptation
27	Glycerolipid metabolism	173 (0.71%)	ko00561	Metabolism	Lipid metabolism
28	Regulation of autophagy	170 (0.7%)	ko04140	Cellular Processes	Transport and catabolism
29	Phagosome	166 (0.68%)	ko04145	Cellular Processes	Transport and catabolism
30	ABC transporters	165 (0.68%)	ko02010	Environmental Information Processing	Membrane transport
31	Galactose metabolism	163 (0.67%)	ko00052	Metabolism	Carbohydrate metabolism
32	Pyruvate metabolism	162 (0.67%)	ko00620	Metabolism	Carbohydrate metabolism
33	Cysteine and methionine metabolism	154 (0.63%)	ko00270	Metabolism	Amino acid metabolism
34	Aminoacyl-tRNA biosynthesis	153 (0.63%)	ko00970	Genetic Information Processing	Translation
35	Glutathione metabolism	152 (0.63%)	ko00480	Metabolism	Metabolism of other amino acids
36	Peroxisome	152 (0.63%)	ko04146	Cellular Processes	Transport and catabolism
37	Carbon fixation in photosynthetic organisms	148 (0.61%)	ko00710	Metabolism	Energy metabolism
38	Nucleotide excision repair	141 (0.58%)	ko03420	Genetic Information Processing	Replication and repair
39	Phosphatidylinositol signaling system	140 (0.58%)	ko04070	Environmental Information Processing	Signal transduction
40	Fatty acid metabolism	130 (0.53%)	ko01212	Metabolism	Global and overview maps
41	Fructose and mannose metabolism	127 (0.52%)	ko00051	Metabolism	Carbohydrate metabolism

42	Terpenoid backbone biosynthesis	125 (0.51%)	ko00900	Metabolism	Metabolism of terpenoids and polyketides
43	Glyoxylate and dicarboxylate metabolism	124 (0.51%)	ko00630	Metabolism	Carbohydrate metabolism
44	DNA replication	119 (0.49%)	ko03030	Genetic Information Processing	Replication and repair
45	Citrate cycle (TCA cycle)	115 (0.47%)	ko00020	Metabolism	Carbohydrate metabolism
46	Sphingolipid metabolism	110 (0.45%)	ko00600	Metabolism	Lipid metabolism
47	Pentose phosphate pathway	109 (0.45%)	ko00030	Metabolism	Carbohydrate metabolism
48	Inositol phosphate metabolism	107 (0.44%)	ko00562	Metabolism	Carbohydrate metabolism
49	Other glycan degradation	107 (0.44%)	ko00511	Metabolism	Glycan biosynthesis and metabolism
50	N-Glycan biosynthesis	107 (0.44%)	ko00510	Metabolism	Glycan biosynthesis and metabolism
51	Mismatch repair	105 (0.43%)	ko03430	Genetic Information Processing	Replication and repair
52	2-Oxocarboxylic acid metabolism	105 (0.43%)	ko01210	Metabolism	Global and overview maps
53	Homologous recombination	104 (0.43%)	ko03440	Genetic Information Processing	Replication and repair
54	Cyanoamino acid metabolism	100 (0.41%)	ko00460	Metabolism	Metabolism of other amino acids
55	Glycine, serine and threonine metabolism	100 (0.41%)	ko00260	Metabolism	Amino acid metabolism
56	Proteasome	100 (0.41%)	ko03050	Genetic Information Processing	Folding, sorting and degradation
57	Lysine degradation	95 (0.39%)	ko00310	Metabolism	Amino acid metabolism
58	Base excision repair	94 (0.39%)	ko03410	Genetic Information Processing	Replication and repair
59	Flavonoid biosynthesis	93 (0.38%)	ko00941	Metabolism	Biosynthesis of other secondary metabolites
60	Porphyrin and chlorophyll metabolism	93 (0.38%)	ko00860	Metabolism	Metabolism of cofactors and vitamins
61	Ascorbate and aldarate metabolism	93 (0.38%)	ko00053	Metabolism	Carbohydrate metabolism
62	Phenylalanine, tyrosine and tryptophan biosynthesis	88 (0.36%)	ko00400	Metabolism	Amino acid metabolism
63	Tyrosine metabolism	88 (0.36%)	ko00350	Metabolism	Amino acid metabolism
64	Basal transcription factors	87 (0.36%)	ko03022	Genetic Information Processing	Transcription
65	Fatty acid elongation	84 (0.35%)	ko00062	Metabolism	Lipid metabolism
66	Protein export	83 (0.34%)	ko03060	Genetic Information Processing	Folding, sorting and degradation
67	Ubiquinone and other terpenoid-quinone biosynthesis	81 (0.33%)	ko00130	Metabolism	Metabolism of cofactors and vitamins
68	alpha-Linolenic acid metabolism	80 (0.33%)	ko00592	Metabolism	Lipid metabolism
69	Arginine and proline metabolism	79 (0.33%)	ko00330	Metabolism	Amino acid metabolism
70	Steroid biosynthesis	78 (0.32%)	ko00100	Metabolism	Lipid metabolism
71	Photosynthesis	78 (0.32%)	ko00195	Metabolism	Energy metabolism
72	Stilbenoid, diarylheptanoid and gingerol biosynthesis	77 (0.32%)	ko00945	Metabolism	Biosynthesis of other secondary metabolites
73	Glycosaminoglycan degradation	73 (0.3%)	ko00531	Metabolism	Glycan biosynthesis and metabolism
74	Ether lipid metabolism	71 (0.29%)	ko00565	Metabolism	Lipid metabolism
75	Alanine, aspartate and glutamate metabolism	69 (0.28%)	ko00250	Metabolism	Amino acid metabolism
76	Sulfur metabolism	69 (0.28%)	ko00920	Metabolism	Energy metabolism
77	Diterpenoid biosynthesis	68 (0.28%)	ko00904	Metabolism	Metabolism of terpenoids and polyketides

78	Valine, leucine and isoleucine degradation	67 (0.28%)	ko00280	Metabolism	Amino acid metabolism
79	Glycosylphosphatidylinositol(GPI)-anchor biosynthesis	66 (0.27%)	ko00563	Metabolism	Glycan biosynthesis and metabolism
80	Flavone and flavonol biosynthesis	65 (0.27%)	ko00944	Metabolism	Biosynthesis of other secondary metabolites
81	Zeatin biosynthesis	63 (0.26%)	ko00908	Metabolism	Metabolism of terpenoids and polyketides
82	Fatty acid biosynthesis	63 (0.26%)	ko00061	Metabolism	Lipid metabolism
83	Propanoate metabolism	63 (0.26%)	ko00640	Metabolism	Carbohydrate metabolism
84	Phenylalanine metabolism	61 (0.25%)	ko00360	Metabolism	Amino acid metabolism
85	Pantothenate and CoA biosynthesis	61 (0.25%)	ko00770	Metabolism	Metabolism of cofactors and vitamins
86	Arginine biosynthesis	61 (0.25%)	ko00220	Metabolism	Amino acid metabolism
87	Sesquiterpenoid and triterpenoid biosynthesis	57 (0.23%)	ko00909	Metabolism	Metabolism of terpenoids and polyketides
88	Tryptophan metabolism	56 (0.23%)	ko00380	Metabolism	Amino acid metabolism
89	Carotenoid biosynthesis	56 (0.23%)	ko00906	Metabolism	Metabolism of terpenoids and polyketides
90	Glycosphingolipid biosynthesis - ganglio series	54 (0.22%)	ko00604	Metabolism	Glycan biosynthesis and metabolism
91	Cutin, suberine and wax biosynthesis	54 (0.22%)	ko00073	Metabolism	Lipid metabolism
92	beta-Alanine metabolism	52 (0.21%)	ko00410	Metabolism	Metabolism of other amino acids
93	Nitrogen metabolism	51 (0.21%)	ko00910	Metabolism	Energy metabolism
94	Limonene and pinene degradation	50 (0.21%)	ko00903	Metabolism	Metabolism of terpenoids and polyketides
95	SNARE interactions in vesicular transport	48 (0.2%)	ko04130	Genetic Information Processing	Folding, sorting and degradation
96	Fatty acid degradation	47 (0.19%)	ko00071	Metabolism	Lipid metabolism
97	Nicotinate and nicotinamide metabolism	47 (0.19%)	ko00760	Metabolism	Metabolism of cofactors and vitamins
98	Arachidonic acid metabolism	46 (0.19%)	ko00590	Metabolism	Lipid metabolism
99	Biosynthesis of unsaturated fatty acids	46 (0.19%)	ko01040	Metabolism	Lipid metabolism
100	Butanoate metabolism	46 (0.19%)	ko00650	Metabolism	Carbohydrate metabolism
101	Folate biosynthesis	41 (0.17%)	ko00790	Metabolism	Metabolism of cofactors and vitamins
102	Valine, leucine and isoleucine biosynthesis	39 (0.16%)	ko00290	Metabolism	Amino acid metabolism
103	Isoquinoline alkaloid biosynthesis	38 (0.16%)	ko00950	Metabolism	Biosynthesis of other secondary metabolites
104	Tropane, piperidine and pyridine alkaloid biosynthesis	36 (0.15%)	ko00960	Metabolism	Biosynthesis of other secondary metabolites
105	Thiamine metabolism	35 (0.14%)	ko00730	Metabolism	Metabolism of cofactors and vitamins
106	One carbon pool by folate	35 (0.14%)	ko00670	Metabolism	Metabolism of cofactors and vitamins
107	Linoleic acid metabolism	34 (0.14%)	ko00591	Metabolism	Lipid metabolism
108	Selenocompound metabolism	30 (0.12%)	ko00450	Metabolism	Metabolism of other amino acids
109	Isoflavonoid biosynthesis	28 (0.12%)	ko00943	Metabolism	Biosynthesis of other secondary metabolites
110	Photosynthesis - antenna proteins	25 (0.1%)	ko00196	Metabolism	Energy metabolism
111	Biotin metabolism	25 (0.1%)	ko00780	Metabolism	Metabolism of cofactors and vitamins
112	Brassinosteroid biosynthesis	23 (0.09%)	ko00905	Metabolism	Metabolism of terpenoids and polyketides
113	C5-Branched dibasic acid	22 (0.09%)	ko00660	Metabolism	Carbohydrate metabolism

	metabolism				
114	Vitamin B6 metabolism	22 (0.09%)	ko00750	Metabolism	Metabolism of cofactors and vitamins
115	Histidine metabolism	21 (0.09%)	ko00340	Metabolism	Amino acid metabolism
116	Degradation of aromatic compounds	18 (0.07%)	ko01220	Metabolism	Global and overview maps
117	Sulfur relay system	17 (0.07%)	ko04122	Genetic Information Processing	Folding, sorting and degradation
118	Glycosphingolipid biosynthesis - globo series	17 (0.07%)	ko00603	Metabolism	Glycan biosynthesis and metabolism
119	Other types of O-glycan biosynthesis	16 (0.07%)	ko00514	Metabolism	Glycan biosynthesis and metabolism
120	Lysine biosynthesis	15 (0.06%)	ko00300	Metabolism	Amino acid metabolism
121	Non-homologous end-joining	15 (0.06%)	ko03450	Genetic Information Processing	Replication and repair
122	Monobactam biosynthesis	15 (0.06%)	ko00261	Metabolism	Biosynthesis of other secondary metabolites
123	Taurine and hypotaurine metabolism	12 (0.05%)	ko00430	Metabolism	Metabolism of other amino acids
124	Synthesis and degradation of ketone bodies	12 (0.05%)	ko00072	Metabolism	Lipid metabolism
125	Indole alkaloid biosynthesis	11 (0.05%)	ko00901	Metabolism	Biosynthesis of other secondary metabolites
126	Riboflavin metabolism	10 (0.04%)	ko00740	Metabolism	Metabolism of cofactors and vitamins
127	Glucosinolate biosynthesis	9 (0.04%)	ko00966	Metabolism	Biosynthesis of other secondary metabolites
128	Lipoic acid metabolism	8 (0.03%)	ko00785	Metabolism	Metabolism of cofactors and vitamins
129	Benzoxazinoid biosynthesis	6 (0.02%)	ko00402	Metabolism	Biosynthesis of other secondary metabolites
130	Monoterpenoid biosynthesis	5 (0.02%)	ko00902	Metabolism	Metabolism of terpenoids and polyketides
131	Anthocyanin biosynthesis	5 (0.02%)	ko00942	Metabolism	Biosynthesis of other secondary metabolites
132	Betalain biosynthesis	3 (0.01%)	ko00965	Metabolism	Biosynthesis of other secondary metabolites
133	Caffeine metabolism	2 (0.01%)	ko00232	Metabolism	Biosynthesis of other secondary metabolites

Table S4. Statistics of DEGs between PH-LF and PH-FB datasets from Formosa lily through KEGG enrichment analysis.

Table S5. Formosa lily unigenes that are homologous with MADS-box -like genes.

Table S6. Primer sets used in this study.

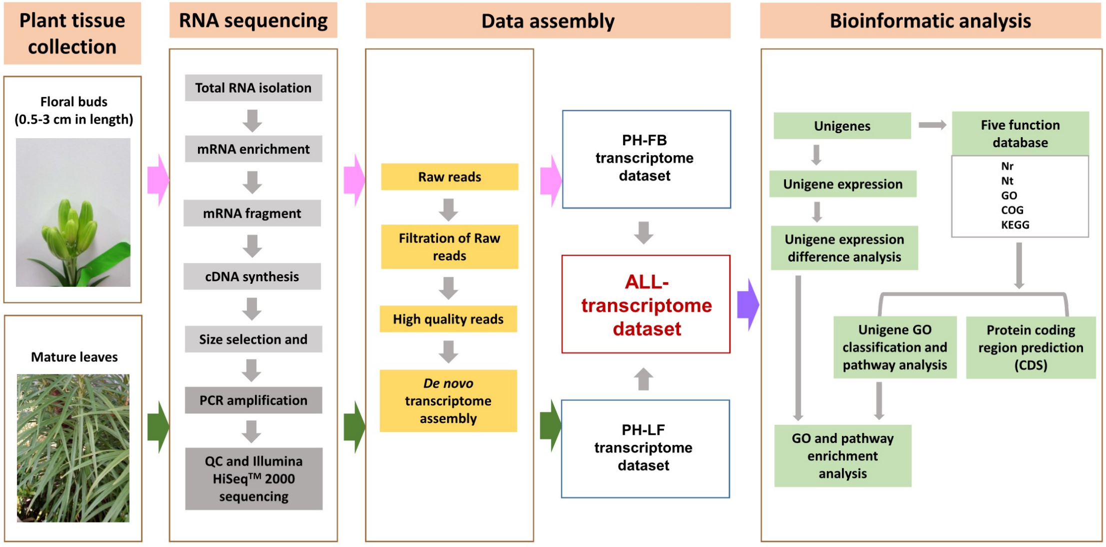

Figure S1. Flowchart of the transcriptome sequencing analysis, including plant tissue collection, RNA sequencing, data assembly, and bioinformatics analysis. The total RNA was extracted from two Formosa lily tissues, namely developing floral buds (0.5–3 cm in length) and mature leaves. Following cDNA preparation of the extracted RNA and sequencing analysis, the retrieved data were used to set up the PH-FB transcriptome dataset for the floral bud tissue and the PH-LF transcriptome dataset for the mature vegetative leaf tissue. Sequences of the unigenes obtained from ALL transcriptome dataset, which was combined from the PH-FB and PH-LF transcriptome datasets, were further analyzed. Finally, differentially expressed unigenes between PH-FB and PH-LF transcriptome datasets were determined.

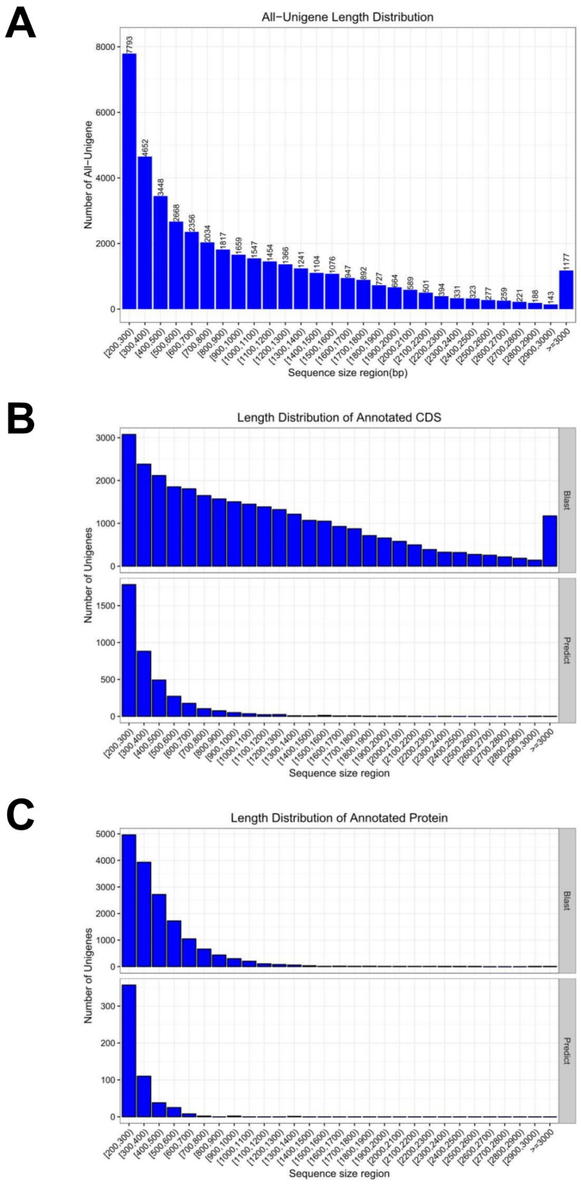

Figure S2. Overview of the sequence reads and assembly of the Formosa lily transcriptome. (A) The size distribution of the ALL-unigenes produced from the ALL transcriptome dataset. (**B**) The size distribution of the CDS produced by searching sequences of ALL-unigenes against various protein databases, including Nr, SWISS-PROT, KEGG, and COG databases, by using the BLASTX program (E-value <10^{-5}). (**C**) The size distribution of the proteins predicted from the CDS of ALL-unigenes. The X-axis indicates the range of unigenes' length. The Y-axis represents the number of assembled unigenes.

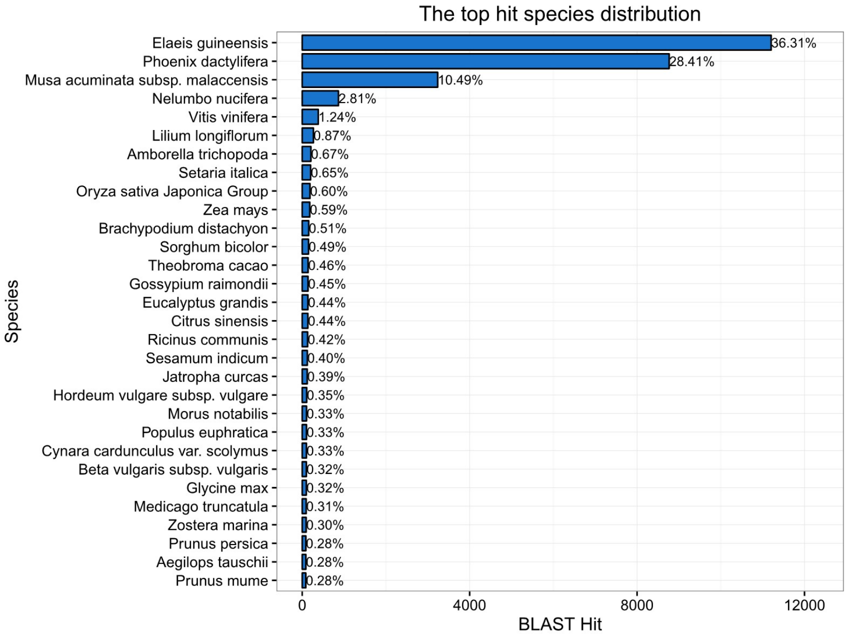

Figure S3. Characteristics of the homology search for the assembled sequences against the Nr database. Species distribution is shown as a percentage of the total homologous sequences with cutoff *E*-value of E ≤ 10^{-5}.

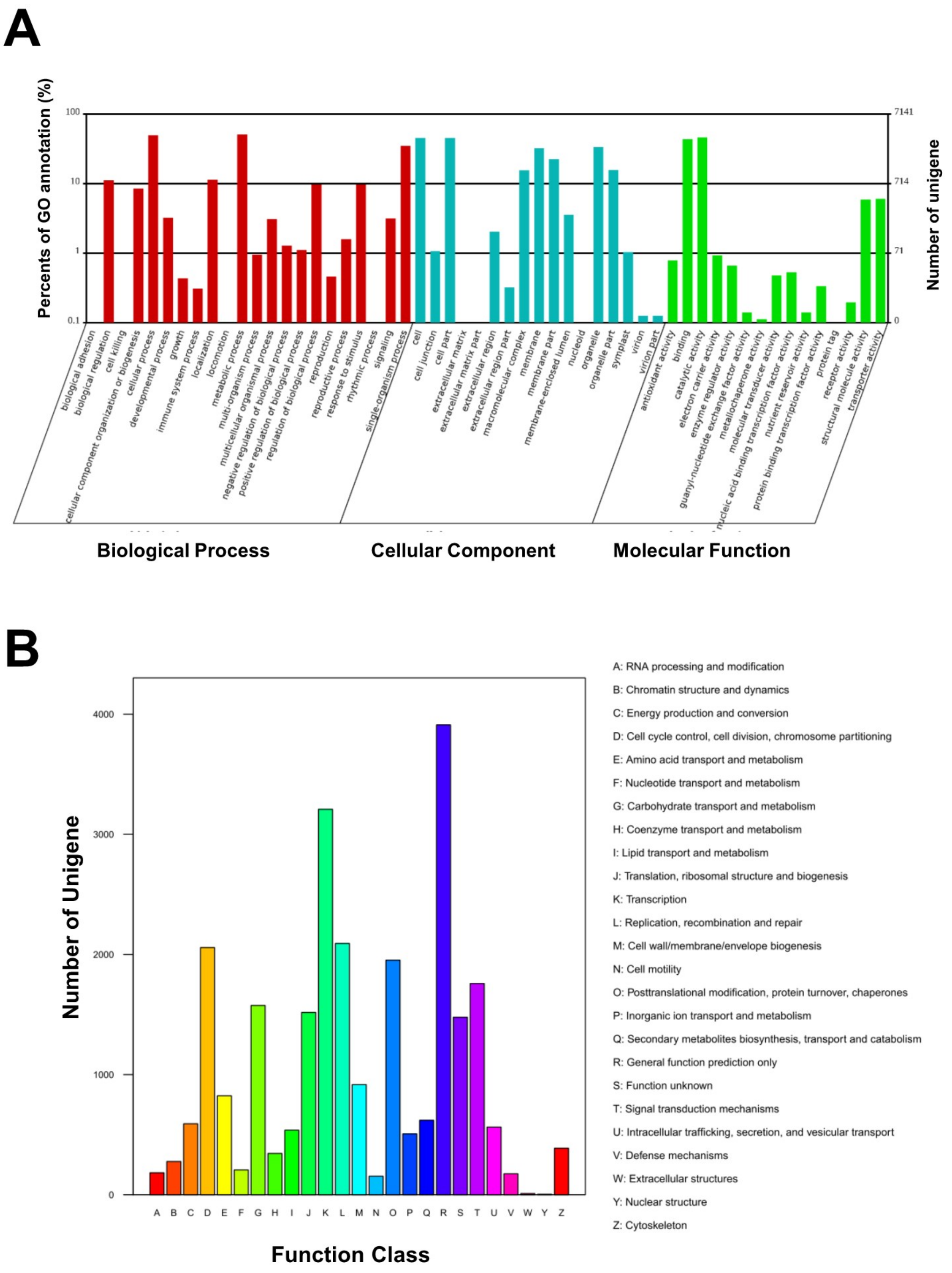

Figure S4. GO and COG classifications of the Formosa lily ALL transcriptome. (**A**) The GO classification of the Formosa lily transcriptome. A total of 7,060 unigenes were assigned into 54 GO annotations classified under three functional categories: Biological Process, Cellular Component, and Molecular Function. (**B**) COG functional classification of the 13,781 unigenes. In total, 25 separate COG categories are represented as A through Z.

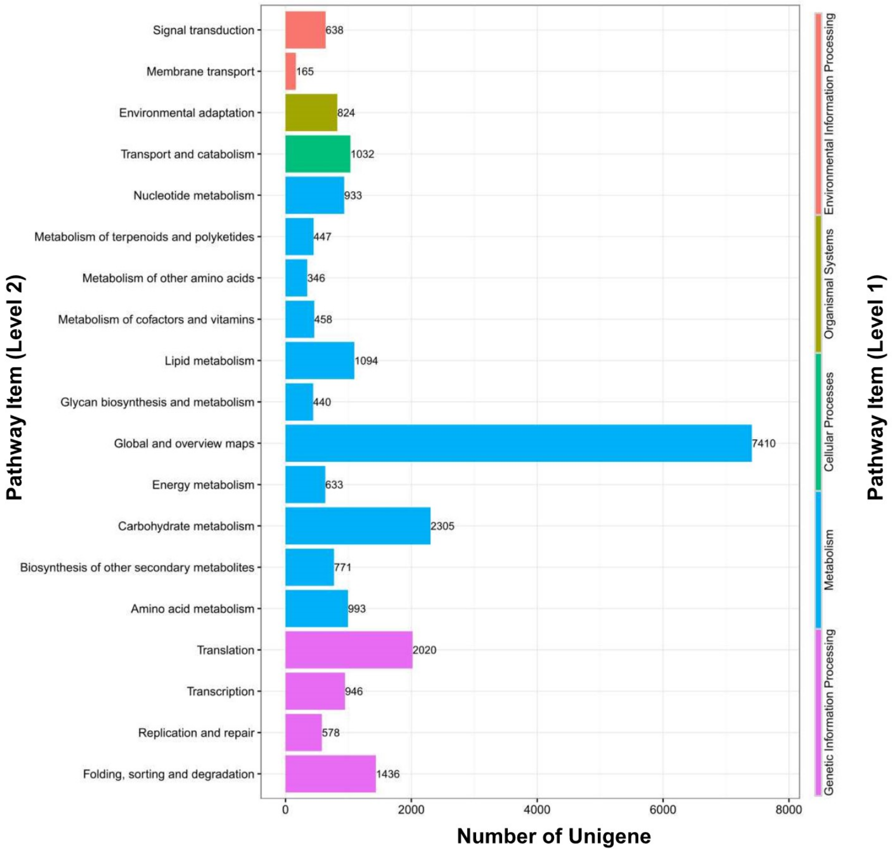

Figure S5. Categorization of Formosa lily unigenes into KEGG pathways and number of unigene per category. The X-axis indicates the number of unigenes corresponding to level 2 of different pathways. The Y-axis exhibits the sub-pathways (level 2) that are classified under the five level 1 pathways, including Genetic information processing, Metabolism, Cellular processes, Organismal systems and Environmental information processing (Table S3).

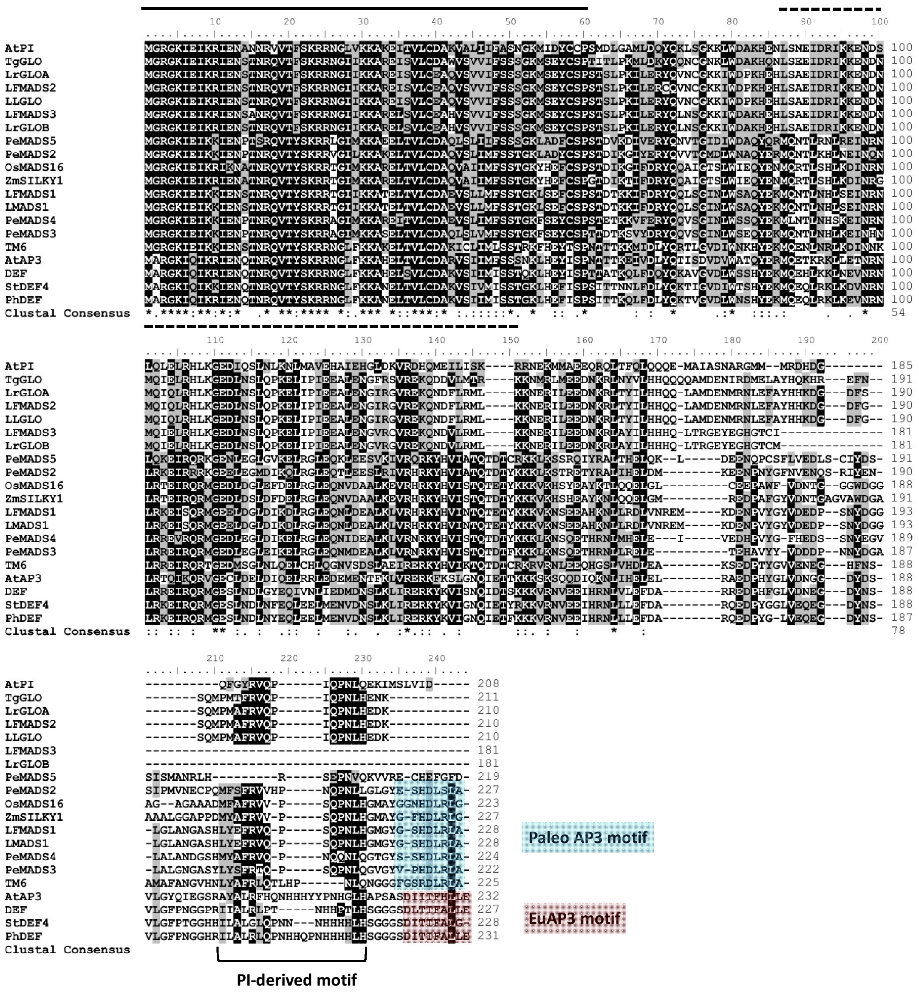

Figure S6. Alignment of the amino acid sequences of LFMADS1, LFMADS2, LFMADS3 and B-class MADS-box orthologs. The protein sequences of B-class MADS-box related proteins aligned in this study were retrieved from NCBI. The sequence alignment was performed by using the ClustalX 1.8 and BioEdit 7.0 softwares. Completely and partially conserved amino acids in the aligned proteins are shaded in black and gray, respectively. The letters and marks in the alignment represent as follows: MADS-box domain (solid line in black), K-box domain (dotted line in black), PI-derived motif (underline in bracket), paleo AP3 motif (highlighted in light blue), and EuAP3 motif (highlighted in red). The names and accession numbers of the respective proteins are given behind the corresponding names of plant species as follows: *Arabidopsis thaliana* [AtPI (NP_191002) and AtAP3 (NP_197524)], *Tulipa gesneriana* [TgGLO (BAC75972)], *Lilium regale* [LrGLOA (BAB91551) and LrGLOB (BAB91552)], *Lilium longiforum* [LLGLO (ABD92703) and LMADS1 (AAM27456)], *Phalaenopsis equestris* [PeMADS2 (AAR26628), PeMADS3 (AAR26629), PeMADS4 (AAR26626), and PeMADS5 (AAR26630)], *Petunia* x *hybrida* [PhDEF (AAQ72510) and TM6 (AAS46017)], *Solanum tuberosum* [StDEF4 (CAA47846)], *Antirrhinum majus* [DEF (CAA44629)], *Zea mays* [ZmSILKY1 (NP_001104951)], and *Oryza sativa* [OsMADS16 (Q944S9)].

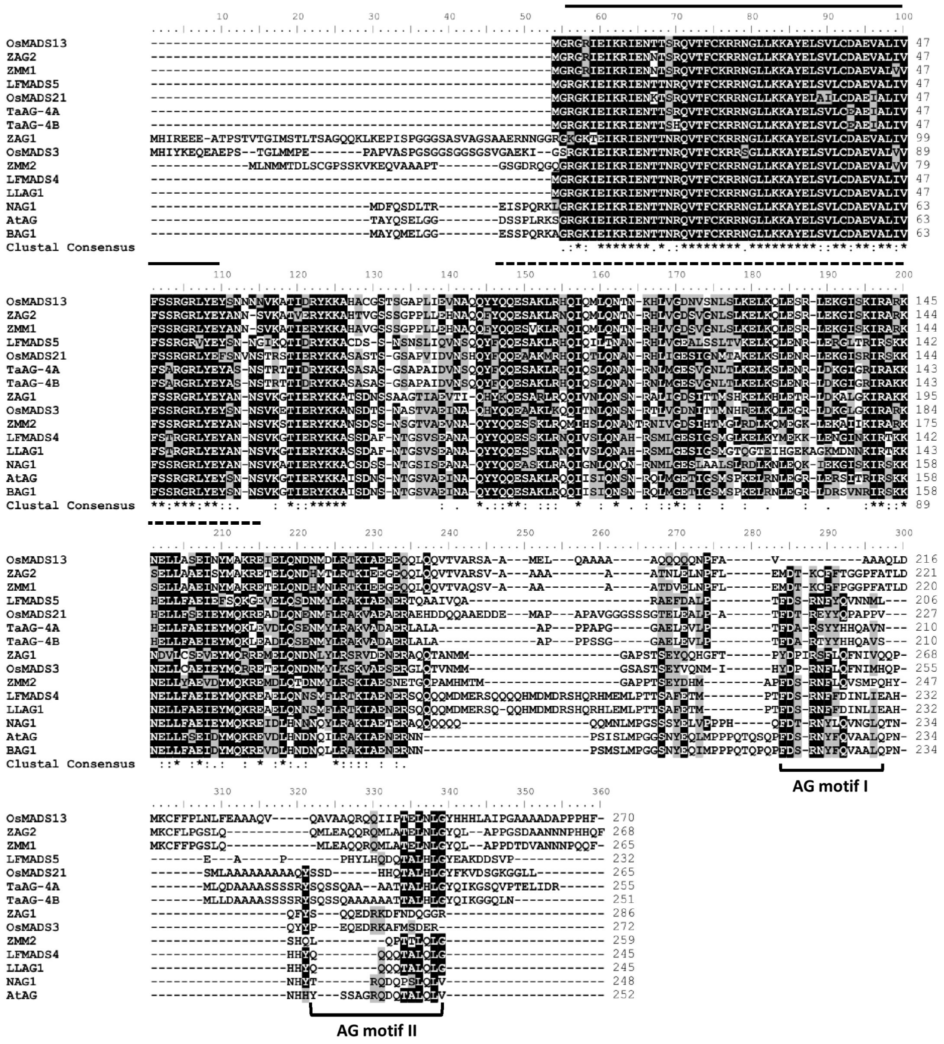

Figure S7. Alignment of the amino acid sequences of LFMADS4, LFMADS5, and C/D-class MADS-box orthologs. The protein sequences of C/D-class MADS-box related proteins aligned in this study were retrieved from NCBI. The sequence alignment was carried out by ClustalX 1.8 and BioEdit 7.0 softwares. Completely and partially conserved amino acids in the aligned proteins are shaded in black and gray, respectively. The letters and marks in the alignment indicate as follows: MADS-box domain (solid line in black), K-box domain (dotted line in black), AG motif I and AG motif II (underline in bracket). The names and accession numbers of the respective proteins are given behind the corresponding names of plant species as follows: *Arabidopsis thaliana* [AtAG (NP_567569)], *Triticum aestivum* L. [TaAG-4A (CAM59072) and TaAG-4B (CAM59073)], *Lilium longiforum* [LLAG1 (AAR98731)], *Brassica napus* [BAG1 (AAA32985)], *Nicotiana tabacum* [NAG1 (AAA17033)], *Zea mays* [ZAG1 (AAA02933), ZAG2 (AAA85870), ZMM1 (CAA57073) and ZMM2 (NP_001105946)], and *Oryza sativa* [OsMADS3 (Q40704), OsMADS13 (Q944S9) and OsMADS21 (XP_015621162)].

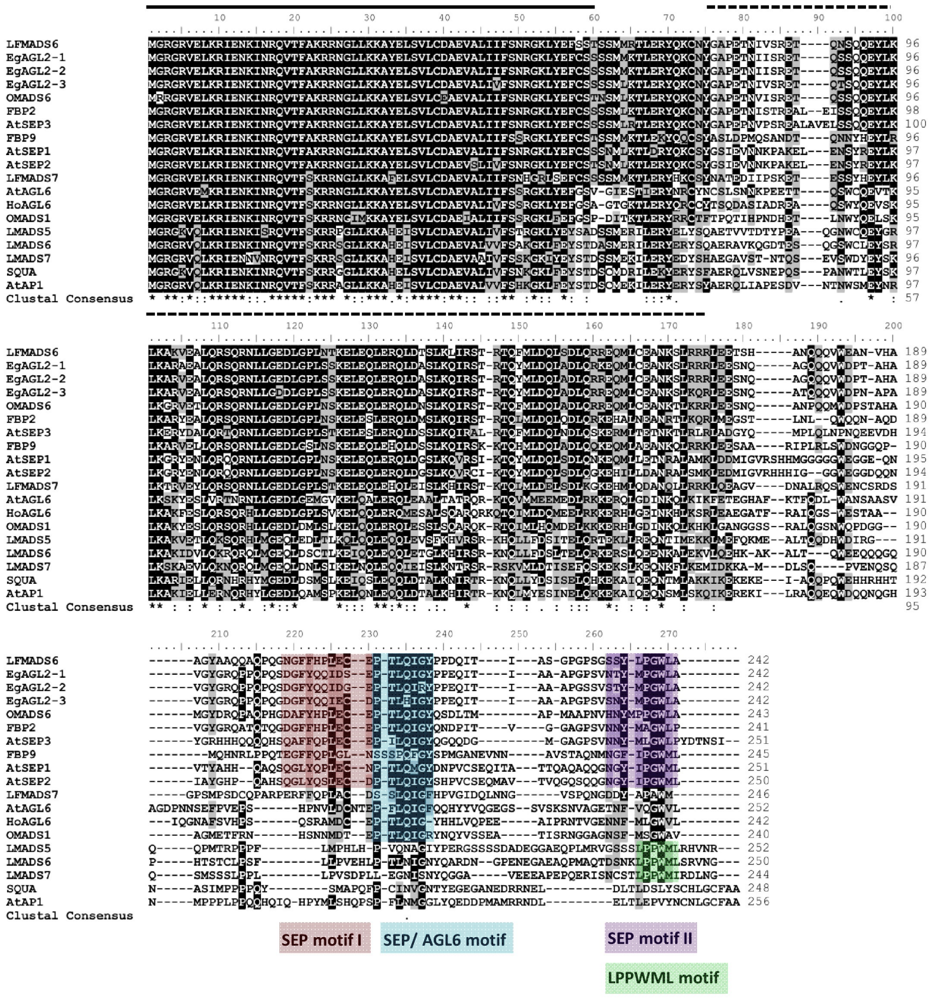

Figure S8. Alignment of the amino acid sequences of LFMADS6, LFMADS7, and E-class MADS-box orthologs. The protein sequences of A/E-class MADS-box related proteins aligned in this study were retrieved from NCBI. The sequence alignment was performed by ClustalX 1.8 and BioEdit 7.0 softwares. Completely and partially conserved amino acids in the aligned proteins are shaded in black and gray, respectively. The letters and marks in the alignment represent as follows: MADS-box domain (solid line in black), K-box domain (dotted line in black), SEP/AGL6 motif (highlighted in light blue), SEP motif I (highlighted in red), SEP motif II (highlighted in purple), and LPPWML motif (highlighted in green). The names and accession numbers of the respective proteins are given behind the corresponding names of plant species as follows: *Arabidopsis thaliana* [AtAP1 (NP_177074), AtSEP1 (NP_568322), AtSEP2 (NP_186880), AtSEP3 (NP_564214) and AtAGL6 (NP_182089)], *Hyacinthus orientalis* [HoAGL6 (AAT88088)], *Petunia hybrida* [FBP2 (Q03489) and FBP9 (AAK21249)], *Lilium longiforum* [LMADS5 (ADT78582), LMADS6 (ADT78583) and LMADS7 (ADT78584)], *Elaeis guineensis* [EgAGL2-1 (XP_010917558), EgAGL2-2 (NP_001306837) and EgAGL2-3 (XP_010913017)], *Antirrhinum majus* [SQUA (CAA45228)], and *Oncidium Gower Ramsey* [OMADS1 (ADJ67237) and OMADS6 (ADJ67238)].

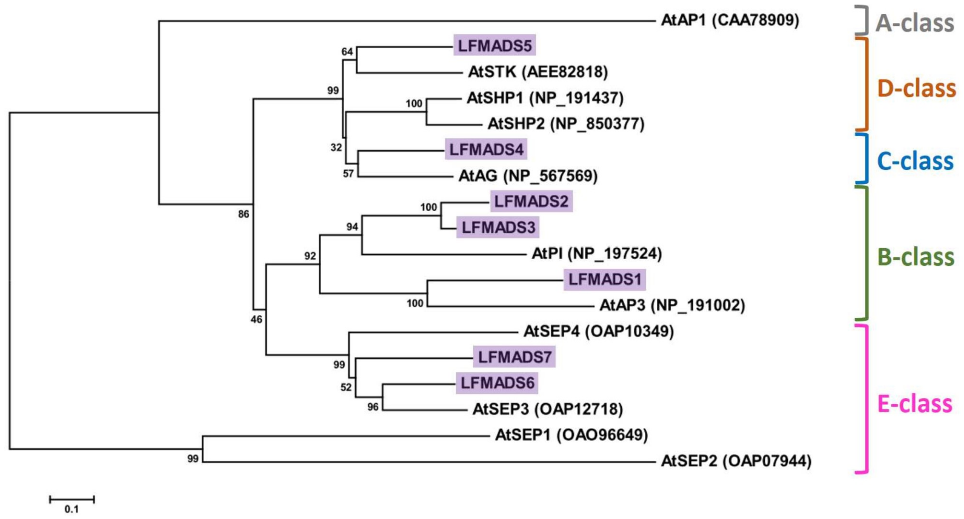

Figure S9. Phylogenetic tree of Formosa lily and *Arabidopsis*. The phylogenetic tree was generated with the neighbor-joining algorithm and evaluated by bootstrap analysis (MEGA version 6.0). Numbers on major branches indicate bootstrap percentage for 1,000 replicates. Subfamilies of the *Arabidopsis* MADS-box genes and the functional classification according to the A-, B-, C-, D- and E-classes are indicated at the right margin.

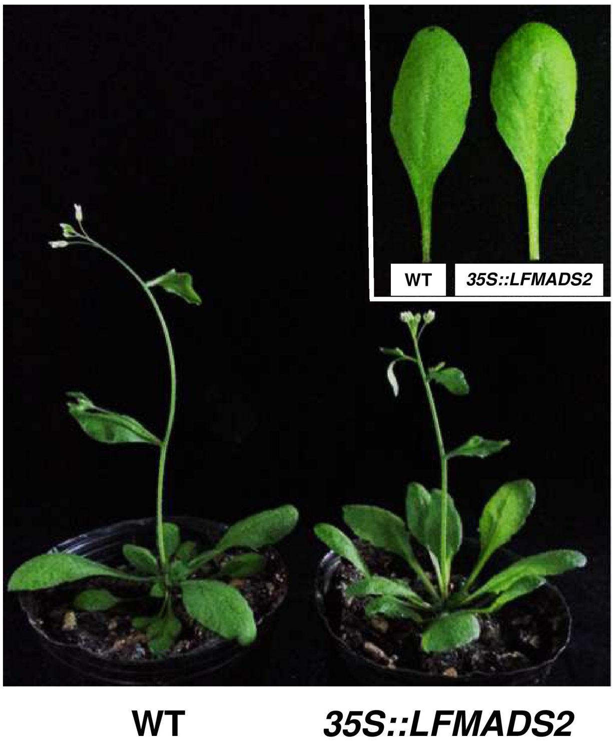

Figure S10. Phenotypic analysis of wild-type (WT) and *35S::LFMADS2* transgenic *Arabidopsis* plants. No significant phenotypic differences between WT and *35S::LFMADS2* transgenic lines, including flowering time, vegetative and floral morphology were observed.

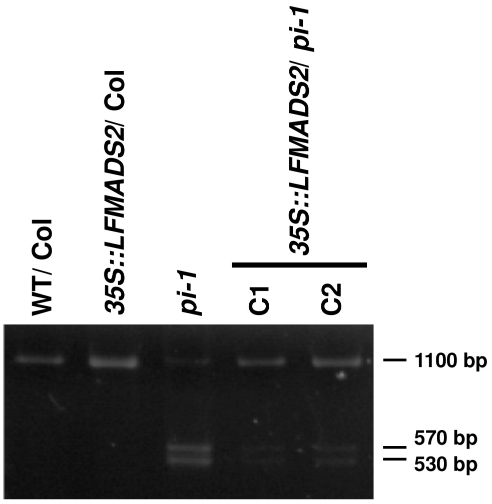

Figure S111. Confirmation of *pi-1* mutants overexpressing *LFMADS2* in F_2 lines (C1 and C2). The specific primer sets (Table S6) were designed to partially amplify respective fragments from both *PI* and *pi* genomic DNAs. The amplified genomic PCR products (1.1 Kb) from wild-type Columbia (Col) ecotype (*PI*/*PI* background) were not digested by BsrI, while the amplified product from *pi-1* (*pi*/*pi* background) genotype obtained from Landsberg *erecta* ecotype could be digested by *Bsr*I, resulting in 570 bp and 530 bp fragments. From the left to right: lane 1, wild-type (*PI*/*PI* background); lane 2, *35S::LFMADS2* transgenic line (*PI*/*PI* background); lane 3, *pi-1* (*pi*/*pi* background); lanes 4 and 5, *35S::LFMADS2* transgenic lines C1 and C2 (*PI*/*pi* background), respectively. As shown here, the amplified PCR products from *35S::LFMADS2* transgenic lines C1 and C2 were partially digested by *BsrI*, and both 570 bp and 530 bp fragments were visible.

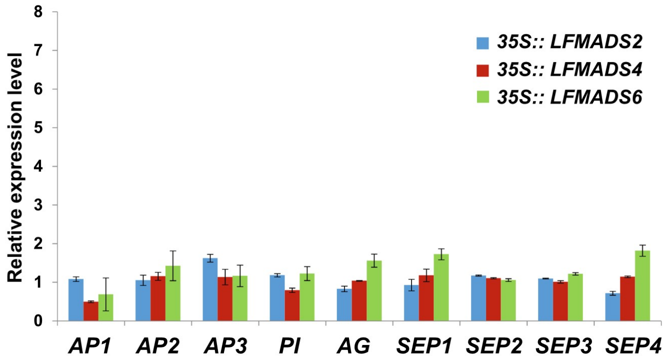

Figure S12. Detection of transcripts for endogenous floral organ-identity genes in transgenic *Arabidopsis* overexpressing *LFMADS2*, *LFMADS4* and *LFMADS6*, respectively. Relative transcription levels of endogenous floral organ-identity genes in *35S::LFMADS2*, *35S::LFMADS4*, and *35S::LFMADS6* transgenic *Arabidopsis* were assessed by real-time qPCR analysis. No obvious differences between wild-type and transgenic lines were detected. Each bar represents the average of three replicates and the standard deviation for each bar is shown. All expression levels of genes were normalized against *TUB2* expression. Primers used in qPCR reactions are listed in Table S6.
